# Molecular basis of senescence transmitting in the population of human endometrial stromal cells

**DOI:** 10.18632/aging.102441

**Published:** 2019-11-05

**Authors:** Anastasiia Griukova, Pavel Deryabin, Alla Shatrova, Elena Burova, Valeria Severino, Annarita Farina, Nikolay Nikolsky, Aleksandra Borodkina

**Affiliations:** 1 Department of Intracellular Signaling and Transport, Institute of Cytology of the Russian Academy of Sciences, Petersburg 194064, Russia; 2 Department of Medicine, University Medical Center (CMU), Faculty of Medicine, Geneva University, Geneva CH-1211, Switzerland

**Keywords:** endometrial stromal cells, senescence, SASP, PAI-1

## Abstract

Hormone-regulated proliferation and differentiation of endometrial stromal cells (ESCs) determine overall endometrial plasticity and receptivity to embryos. Previously we revealed that ESCs may undergo premature senescence, accompanied by proliferation loss and various intracellular alterations. Here we focused on whether and how senescence may be transmitted within the ESCs population. We revealed that senescent ESCs may induce paracrine senescence in young counterparts via cell contacts, secreted factors and extracellular vesicles. According to secretome-wide profiling we identified plasminogen activator inhibitor -1 (PAI-1) to be the most prominent protein secreted by senescent ESCs (data are available via ProteomeXchange with identifier PXD015742). By applying CRISPR/Cas9 techniques we disclosed that PAI-1 secreted by senescent ESCs may serve as the master-regulator of paracrine senescence progression within the ESCs population. Unraveled molecular basis of senescence transduction in the ESCs population may be further considered in terms of altered endometrial plasticity and sensitivity to invading embryo, thus contributing to the female infertility curing.

## INTRODUCTION

Cellular senescence is a unique fate of proliferating cells. The functional meaning of this reaction is to prevent propagation of cells bearing damages, which is implemented by the senescence-associated proliferation arrest [1, 2]. Preserved synthetic activity along with malfunctioning of intracellular systems result in a gradual switch of the secretory profile of senescent cells, termed the senescence-associated secretory phenotype (SASP) [3]. SASP includes a variety of signaling molecules, such as pro-inflammatory cytokines, chemokines, growth factors, matrix metalloproteases and serine proteases, tissue inhibitors of metalloproteases (TIMP), insulin-like growth factor binding proteins (IGFBPs), and reactive oxygen species (ROS) [1, 4, 5]. More recently, extracellular vesicles (EV) were recognized as the essential SASP components [6, 7].

SASP is the way senescent cells communicate with their surroundings. Factors composing SASP may be transmitted through cell contacts directly to the adjacent cells or may act distantly (via soluble factors and EV), remodeling extracellular matrix and altering overall functioning of the surrounding microenvironment [5, 8, 9]. Therefore, SASP predetermines various consequences of the existence of senescent cells within tissues. Strictly speaking, the basic biological meaning of SASP is to attract phagocytic immune cells by creating a strong pro-inflammatory microenvironment. Engaged immune cells are responsible for the removal of senescent ones, thus preserving normal tissue functioning. However, depending on the duration of SASP secretion and the state of the surrounding cells, these pro-inflammatory factors along with other SASP components may mediate various outcomes. For instance, SASP components may stimulate the proliferation of the surrounding cells, may accelerate wound closure by inducing myofibroblast differentiation, thus promoting tissue repair [10–12]. At the same time factors secreted by senescent cells can enhance proliferation, survival, and epithelial-to-mesenchymal transition in both committed pre-neoplastic and cancer cells harbored in the tissue, reflecting tumor-promoting role of SASP [4, 13, 14]. Another outcome of the altered paracrine activity of senescent cells is that SASP may sensitize normal neighboring cells to senesce, accelerating propagation of senescence within cell population and contributing to tissue dysfunction [15]. Although there are several common factors secreted by various types of senescent cells, the precise composition of SASP may significantly vary according to cell types and senescence triggers. Both cell-dependent variations in the SASP composition and type/state of the neighboring cells may determine different impacts of senescent cells on the surrounding microenvironment leading to diverse consequences. Therefore, it is expedient to assess SASP functions for concrete producing and target cell types.

Being a critical component of stromal compartment of endometrium lining uterus, endometrial stromal cells (ESCs) have important functions in the context of female reproduction. ESCs are capable for proliferation and tissue-specific differentiation, mediating both cyclic restoration of the functional layer of endometrium and hormone-induced decidualization of endometrial stroma required for embryo implantation [16, 17]. In our previous findings we have clearly shown that premature senescence is the primary reaction of ESCs in response to various stresses [18–20]. Senescent ESCs display most of the typical features of cellular senescence, e.g. irreversible cycle block, proliferation loss, decreased differentiation and migration ability, enhanced SA-β-Gal activity, hypertrophy, increased ROS levels and so on [18, 21, 22]. The fact that ESCs may senesce in response to stress factors and, therefore lose proliferation and differentiation abilities, is itself significant for normal functioning of endometrial tissue. However, changes of secretory profile during ESCs senescence may have even more crucial aftermaths in terms of female fertility. On the one hand, senescent cells via SASP may transduce damage on the adjacent normal ESCs, leading to senescence propagation and reduced endometrial plasticity. On the other hand, factors secreted by senescent ESCs may interfere the fine-tuned dialog between endometrium and invading embryo. Both consequences of SASP action may lead to implantation failures and pregnancy complications. Taking into consideration an obvious biomedical significance, the present study aimed to perform the comprehensive analysis of the secretome profile of senescent ESCs, and to reveal molecular basis of how senescent cells and their SASP affect normal ESCs.

## RESULTS

### Co-culturing with senescent ESCs negatively affects their young counterparts

**Figure 1:**
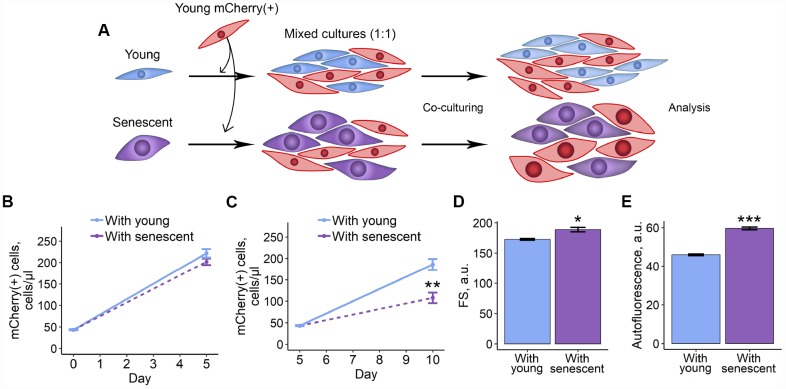
**2D co-culturing with senescent ESCs negatively affects surrounding cells.** (**A**) Experimental scheme of co-culturing of young mCherry-labeled ESCs with unlabeled young or senescent ones in 2D condition. (**B** and **C**) Growth curves of mCherry-labeled ESCs (co-cultured either with young or senescent cells) before and after reseeding, respectively. Cell number was determined by FACS at the indicated time points. (**D**) and (**E**) Cell size and autofluorescence of mCherry-labeled ESCs measured by FACS after 10 d of co-culturing. Forward scatter (FS) reflects the average cell size. Values are M ± S.D. (N=3). * – p<0.05, ** – p<0.01, *** – p<0.005 by Student’s t-test.

In order to test whether senescent ESCs may somehow modulate properties of their young surroundings, firstly, we examined the effects of co-culturing of senescent ESCs with young cells. To induce senescence we applied treatment design well described in our previous studies [18, 21, 22]. In brief, ESCs were treated with sublethal H_2_O_2_ dose and additionally cultured for 7 days until reaching irreversible senescence. Notably, in 7 days after the oxidative stress ESCs displayed most of the classical senescence features. In order to distinguish between senescent and young cells, the latest were transduced with lentiviruses (LV) encoding mCherry fluorescent protein. We next seeded unlabelled young or senescent cells in 1:1 ratio with mCherry-expressing young ESCs (Figure 1A). Cells were co-cultured for 5 days, then reseeded and additionally cultured for the indicated time followed by the estimation of various parameters of mCherry-positive cells. As shown in Figure 1B–1D, co-culturing with senescent ESCs resulted in decreased proliferation rate, increased cell size and autofluorescence levels of mCherry-expressing cells, compared to those co-cultured with young ESCs. Of note, increase in autofluorescence reflects accumulation of lipofuscin – a nondegradable product of protein and lipid oxidation shown to accumulate in senescent cells [23]. These data reveal that senescent ESCs may negatively affect their young microenvironment.

**Figure 2:**
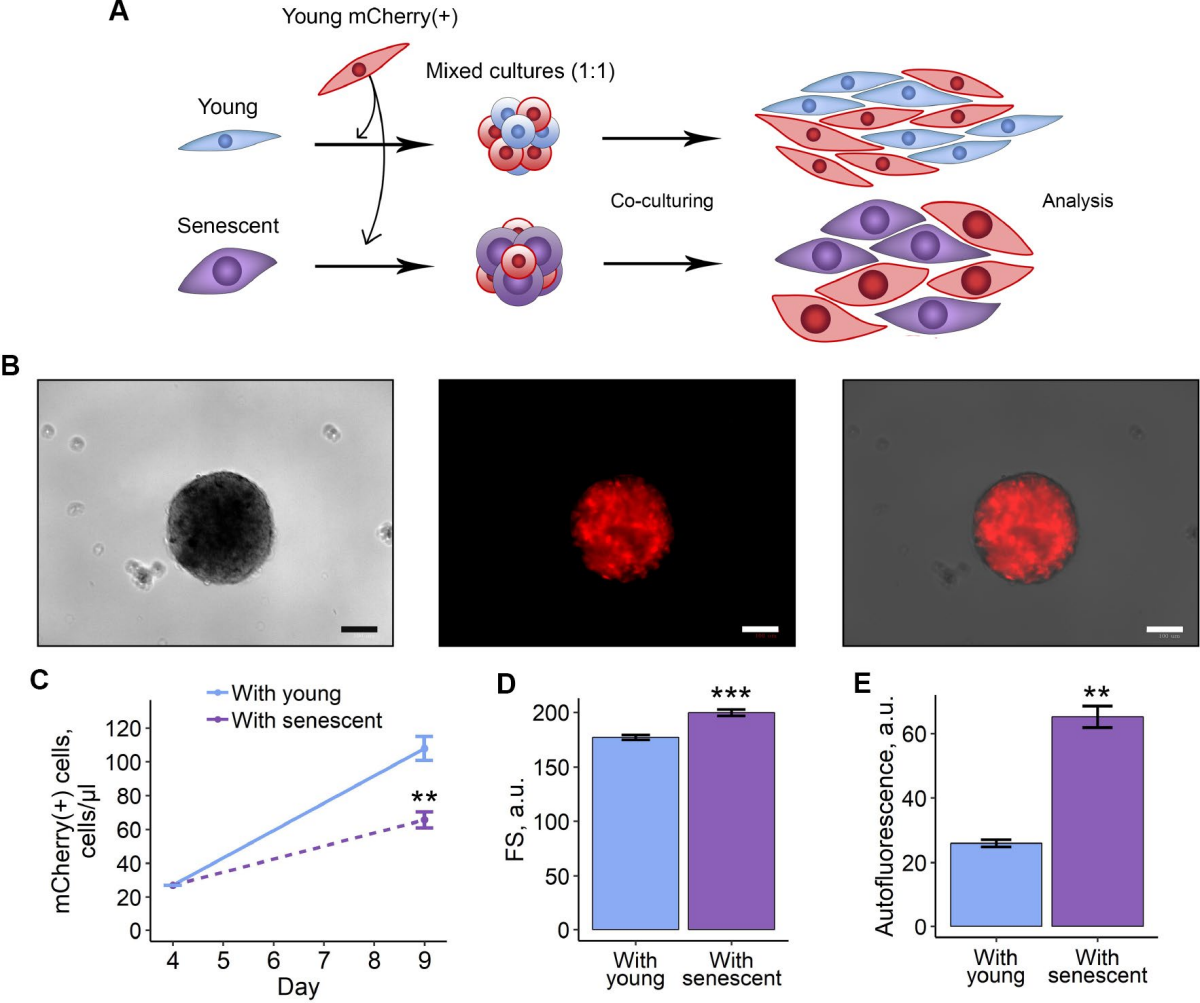
**3D co-culturing with senescent ESCs negatively affects surrounding cells.** (**A**) Experimental scheme of co-culturing of young mCherry-labeled ESCs with unlabeled young or senescent ones in 3D condition. (**B**) Representative photographs of spheroids formed from a mixture of unlabeled and mCherry-labeled ESCs. Scale bars of all images are 500 μm. (**C**–**E**) Growth curves, cell size and autofluorescence of mCherry-labeled ESCs, respectively. Cells were cultured in spheroids during 4 d, trypsinized and cultured for additional 5 d up to analysis by FACS. Forward scatter (FS) reflects the average cell size. Values are M ± S.D. (N=3 for (**C**) and (**D**), N=2 for (**E**)). ** – p<0.01, *** – p<0.005 by Student’s t-test.

In order to verify obtained results, we recapitulated the above co-culture scheme in 3D model. On the one hand, culturing cells in 3D is thought to be more physiologically relevant, and, on the other hand, it allows forming more cell contacts, thus, cell communication should be tighter than in 2D. Here we applied the hanging drop technique to form 3D spheroids that contained mCherry-expressing young ESCs either with unlabelled senescent or young cells (Figure 2A, 2B). Mixed cells were maintained in spheroids for 4 days, and then were dissociated and cultured in monolayer conditions until analysis. As expected, the results previously obtained in 2D co-culturing models completely coincided with those in 3D-models, namely mCherry-positive ESCs co-cultured with senescent cells were characterized by reduced proliferation rate, hypertrophy and enhanced autofluorescence levels (Figure 2C–2E). Of note, co-culturing young and senescent cells in 3D led to even more pronounced increase in cell size and autofluorescence levels of mCherry-positive cells compared to those in 2D.

Together, these findings indicate that senescent ESCs transduce negative impact as bystander effect in surrounding proliferating cells.

### SASP from senescent ESCs triggers senescence in young cells

**Figure 3:**
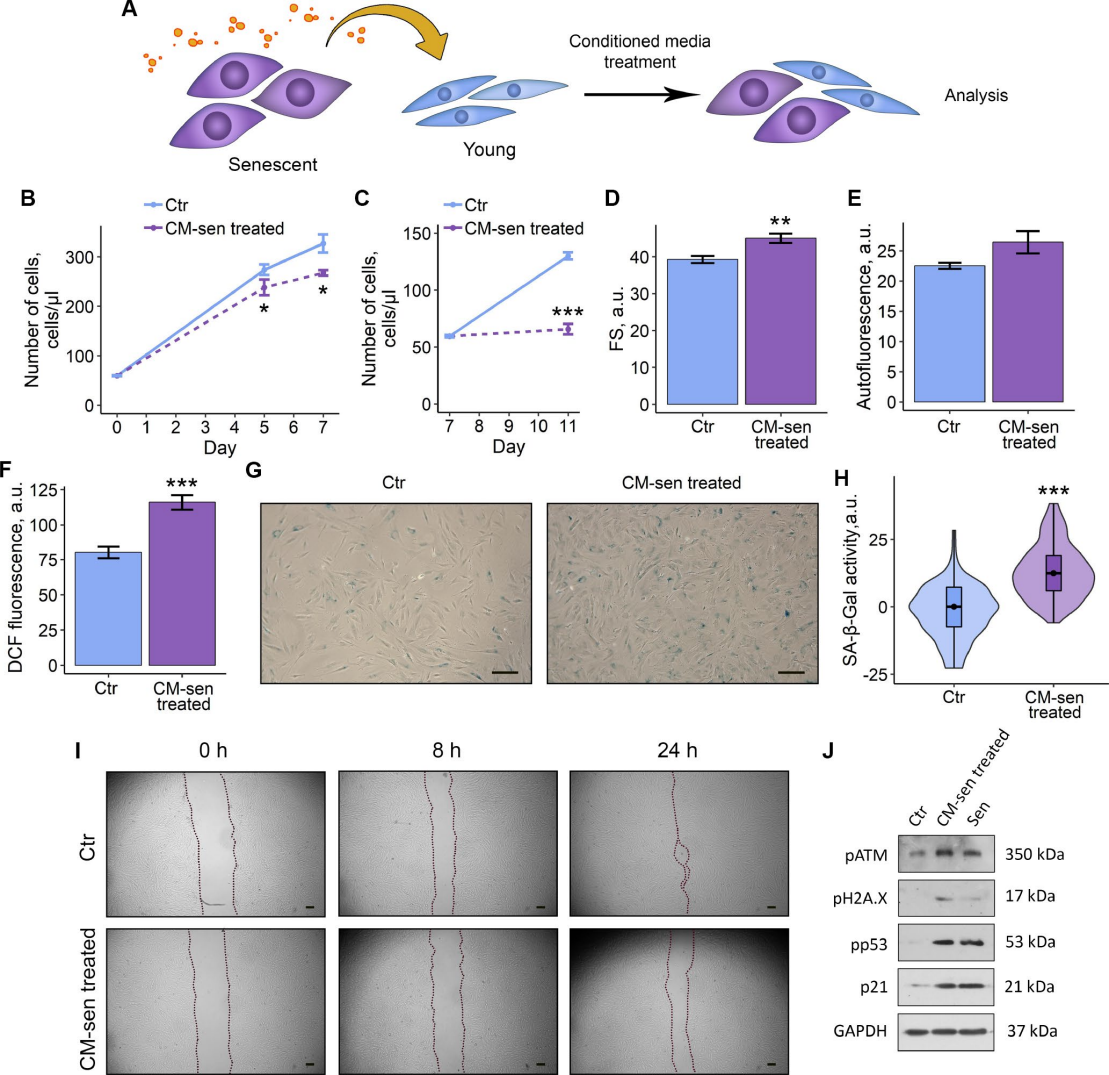
**SASP from senescent ESCs triggers senescence in young cells.** Ctr – young ESCs cultured in standard conditions. CM-sen treated – ESCs exposed to condition medium from senescent cells. Sen – senescent ESCs. (**A**) Experimental scheme of ESCs CM-sen treatment. (**B**) and (**C**) Growth curves of ESCs before and after reseeding, respectively. Cell number was determined by FACS at the indicated time points. (**D**–**F**) Cell size, autofluorescence and intracellular ROS levels of ESCs determined by FACS after 9 d of CM-sen treatment. Forward scatter (FS) reflects the average cell size, DCF fluorescence reflects ROS levels by oxidation of H_2_DCF-DA. Values are M ± S.D. (N=3). * – p<0.05, ** – p<0.01, *** – p<0.005 by Student’s t-test. (**G**) SA-β-Gal staining of Ctr and CM-sen treated ESCs. After 7 d of treatment ESCs were reseeded and additionally cultured for 3 d in order to perform staining of non-confluent cultures. (**H**) Quantification of SA-β-Gal activity values (**G**). Values presented as M and 95 % C.I. (N=100). *** – p<0.005 by Mann-Whitney test. (**I**) Wound healing analysis of ESCs cultured in standard conditions or pre-exposed to CM-sen for 4 d. Cells’ monolayers were scratched and migration activity of cells were estimated at the indicated time points. Scale bars of all images are 500 μm. (**J**) Western blot analysis of ATM, H2A.X and p53 phosphorylation levels and p21 protein expression performed after 7 d of treatment. Representative results of the three experiments are shown in the Figure. GAPDH was used as loading control.

Reduction in proliferation rate, cell hypertrophy and accumulation of lipofuscin revealed in the co-culturing experiments allowed us to suggest that factors produced by senescent ESCs may induce paracrine senescence in the young neighboring cells. To determine whether extracellular factors were endowed with senescence-induction properties, the most typical senescence markers were evaluated in young ESCs cultured in conditioned medium from senescent cells (CM-sen) (Figure 3A). CM-sen was obtained from senescent ESCs in 7 d after the sublethal oxidative stress according to the protocol described in the Experimental procedures section. In line with the results obtained in co-culturing experiments, ESCs cultured in CM-sen were characterized by the proliferation slowdown that was more significant after reseeding, increased cell size, and accumulation of lipofuscin granules, reflected by the increased autofluorescence (Figure 3B–3E).

Further precise investigation of CM-sen treated ESCs revealed other important senescence features, namely elevated intracellular ROS levels, enhanced SA-β-Gal activity and reduced migration capacity (Figure 3F–3I). At the molecular level paracrine senescence of young ESCs induced by SASP factors was associated to the activation of the DNA damage response members – ATM and H2AX, and the following signal transduction via p53/p21 pathway (Figure 3J). Notably, detected phosphorylation levels of ATM, H2AX, p53 and expression level of p21 induced by CM-sen were comparable to those in ESCs aged in response to oxidative stress. These results demonstrate that extrinsic factors secreted by senescent ESCs are able to promote senescence phenomena in the neighboring cells.

### Both soluble factors and extracellular vesicles secreted by senescent ESCs mediate SASP-induced paracrine senescence in young cells

**Figure 4:**
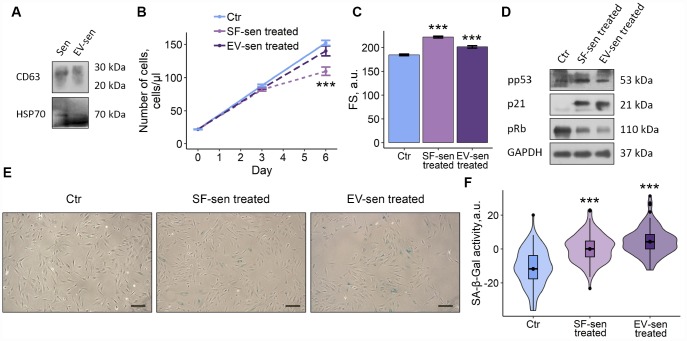
**Soluble factors and extracellular vesicles secreted by senescent ESCs trigger senescence in young cells.** Sen – senescent ESCs. Ctr – young ESCs cultured in standard conditions. SF-sen or EV-sen treated – young ESCs exposed to soluble factors and extracellular vesicles secreted by senescent ESCs, respectively. (**A**) Western blot analysis of CD63 and HSP70 total proteins amount in Sen and EV-sen lysates. (**B**) and (**C**) Growth curves and cell size of Ctr, SF-sen and EV-sen treated ESCs determined by FACS. Forward scatter (FS) reflects the average cell size evaluated after 6 d of exposure. Values are M ± S.D. (N=3). *** – p<0.005 by ANOVA with Tukey HSD versus Ctr. (**D**) Western blot analysis of p53 and Rb phosphorylation levels and p21 protein expression performed after 7 d of treatment. Representative results of the three experiments are shown in the Figure. GAPDH was used as loading control. (**E**) SA-β-Gal staining of Ctr, SF-sen and EV-sen treated ESCs. After 7 d of treatment ESCs were reseeded and additionally cultured for 3 d in order to perform staining of non-confluent cultures. (**H**) Quantification of SA-β-Gal activity values (**E**). Values presented as M and 95 % CI (N=100). *** – p<0.005 by ANOVA with Tukey HSD versus Ctr.

According to one of the existing classifications, factors secreted by senescent cells can be divided into soluble fraction (SF) and extracellular vesicles (EV) [7]. Therefore, we raised a question, what SASP fraction predominantly mediates negative effects of senescent ESCs on young cells. To do so, we applied the most common method of EV purification based on ultracentrifugation that allowed us to separate CM-sen into SF and EV. After serial centrifugations EV fraction obtained from CM-sen was identified by the presence of CD63 and heat shock protein 70, proteins commonly found in EV (Figure 4A).

Having distinguished fractions in CM-sen, we then compared effects of SF and EV secreted by senescent cells on young ESCs. As shown in Figure 3B–3F, both fractions negatively affected the fate of young cells, namely ESCs treatment either with SF or EV both obtained from senescent cells led to reduced proliferation, increased cell size, activated p53/p21/Rb pathway and enhanced SA-β-Gal staining. Notably, the undesirable influence of SF on the tested ESCs parameters was more significant compared to EV, suggesting that SF has primary contribution to the SASP-induced senescence of young ESCs. Nevertheless, according to the described results, both SF and EV secreted by senescent ESCs are responsible for paracrine senescence propagation in the population of young cells.

### Proteomic analysis of ESCs secretome

**Figure 5:**
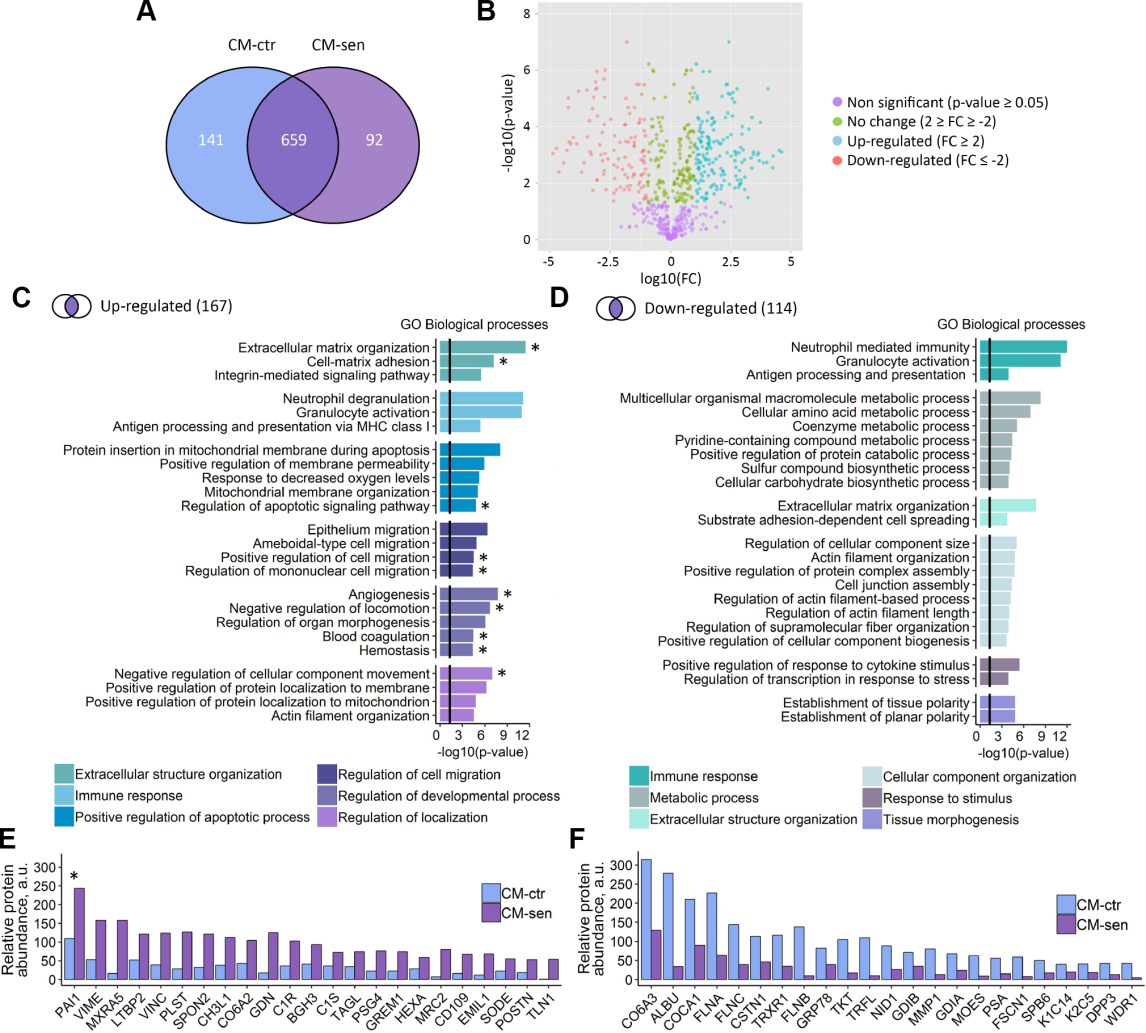
**Proteomic analysis of ESCs secretome.** CM-ctr and CM-sen – conditioned media from young or senescent ESCs, respectively. (**A**) Venn diagram presentation of all peptides identified within CMs by LC-MS/MS. (**B**) Volcano plot of proteins differentially secreted by Ctr and Sen ESCs. (**C**) and (**D**) Functional enrichment analysis in GO BP terms of up- and down-regulated proteins in CM-sen versus CM-ctr. Identified processes are organized in modules based on common parent GO terms presented in legends. To control the false discovery rate (FDR) to correct the p-value the Benjamini method was applied. Black line indicates threshold at p=0.05. (**E**) and (**F**) Levels of top up- and down-regulated proteins in CM-sen versus CM-ctr, respectively. Processes involving PAI-1 are marked with asterisk (*).

Having established the fact that both soluble factors and EVs secreted by senescent ESCs mediate senescence induction in the adjacent young counterparts via paracrine mechanisms, we then focused on the precise analysis of the overall SASP protein content without its fractioning. To identify secreted proteins related to the ESCs senescence we performed a comparative secretome analysis by applying a shotgun proteomic strategy. Proteins from CM obtained from young ESCs (CM-ctr) and CM-sen were identified by liquid chromatography tandem-mass spectrometry (LC-MS/MS) and the following bioinformatic analysis. Globally, 892 proteins were identified (at least 2 unique peptides, 1 % FDR) across both conditions (Figure 5A). Among these, 659 proteins were common to CM-ctr and CM-sen, while 141 and 92 proteins were uniquely detected in CM-ctr and CM-sen, respectively (Figure 4, Supplementary Tables 1, 2).

The quantitative analysis performed by spectral counting allowed the statistically significant quantification of 430 proteins, and revealed 167 up-regulated and 114 down-regulated proteins in the senescent condition (Figure 5B, Supplementary Tables 3, 4). The GO analysis for biological processes of the up-regulated proteins revealed that several enriched processes are involved in the classical SASP response (Figure 5C). In detail, proteins up-regulated in senescent ESCs secretome were involved in extracellular structure organization, immune response, regulation of cell migration and positive regulation of apoptosis. Interestingly, among down-regulated proteins we also revealed proteins involved in extracellular structure organization and immune response, suggesting overall alterations of both processes in senescent ESCs (Figure 5D).

Of note, we observed strong up-regulation of CHI3L1 protein in secretome of senescent ESCs (Figure 5E). This protein belongs to the chitinase gene family. Previously, another member of this family CHI3L3 was described as a secreted marker of DNA damage and senescence [24]. According to the results presented in Figure 5E and 5F the most prominent alterations were detected for Plasminogen activator inhibitor 1 (PAI-1). Moreover, PAI-1 turned out to be involved approximately in half of biological processes positively regulated in CM-sen (Figure 5C, processes involving PAI-1 are marked with asterisk). Therefore, we further focused on the investigation of the possible PAI-1 role in triggering SASP-mediated paracrine senescence in ESCs.

### Alterations of PAI-1 secretion levels modulate SASP-induced senescence propagation within young ESCs population

**Figure 6:**
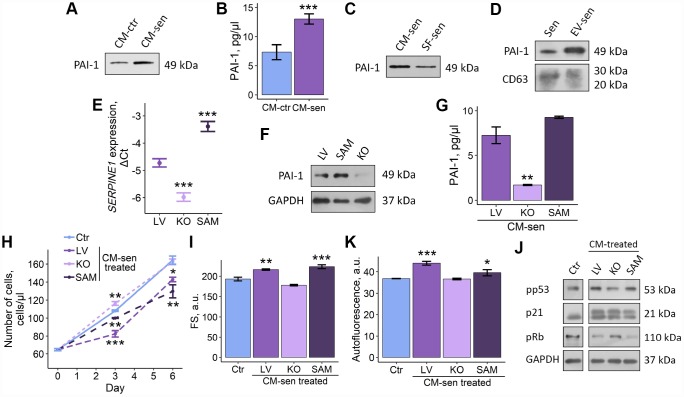
**Altered PAI-1 secretion levels modulate SASP-induced senescence propagation within ESCs population.** Ctr – young ESCs cultured in standard conditions. Sen – senescent ESCs. CM-ctr and CM-sen – conditioned media from young or senescent ESCs, respectively. SF-sen or EV-sen treated – young ESCs exposed to soluble factors and extracellular vesicles secreted by senescent ESCs, respectively. LV, KO and SAM – gene-modified ESCs with unaffected, down-regulated and overexpressed SERPINE1 gene. CM-sen LV, KO, SAM treated – young ESCs exposed to conditioned media from senescent gene-modified cells. (**A, B**) Western blot analysis and ELISA of PAI-1 composition in CM-ctr and CM-sen. For western blot CMs were collected from equal numbers of cells and in equal volumes of media. ELISA values presented as M ± S.D. (N=4). *** – p<0.005 by Student’s t-test. (**C, D**) Western blot of PAI-1 content in SF-sen and EV-sen obtained as described in Experimental procedures section. CD63 was used as EV marker protein. For western blot CMs and SF were collected from equal numbers of cells and in equal volumes of media. (**E**, **F**) PAI-1 expression levels in LV, KO and SAM estimated by RT-PCR and western blot, respectively. Values are M ± S.D. (N=3). *** – p<0.005 by ANOVA with Tukey HSD versus Ctr. (**G**) PAI-1 levels in LV, KO, SAM CM-sen by ELISA. Values are M ± S.D. (N=2). ** – p<0.01 by ANOVA with Tukey HSD versus Ctr. (**H**–**J**) Growth curves, cell size and autofluorescence of Ctr ESCs or LV, KO, SAM CM-sen treated ESCs by FACS. Cell size and autofluorescence after 6 d of treatment. Forward scatter (FS) reflects the average cell size. Values are M ± S.D. (N=3). * – p<0.05, ** – p<0.01, *** – p<0.005 by ANOVA with Tukey HSD versus Ctr of the same time point. (**K**) Western blot analysis of p53 and Rb phosphorylation levels and p21 protein expression performed after 6 d of treatment. Representative results of the three experiments are shown in the Figure. GAPDH was used as loading control.

As the starting point to test possible PAI-1 role in paracrine senescence triggering in young ESCs, we validated results of proteomic analysis by ELISA and Western blotting of CM-ctr and CM-sen samples, using specific antibodies against PAI-1. In line with mass spectrometry data, we revealed significantly increased PAI-1 content in CM obtained from senescent ESCs compared to those from young cells, confirming enhanced PAI-1 secretion by senescent ESCs (Figure 6A, 6B).

We then checked what SASP fraction, SF or EV, contained PAI-1. Interestingly, both SF and EV produced by senescent ESCs were PAI-1 positive (Figure 6C, 6D). Thus, we can speculate that SF or EV alone may replicate negative influence of full SASP on young ESCs due to PAI-1 content.

Further, to claim functional role of PAI-1 secreted by senescent ESCs in paracrine senescence propagation, we modulated expression of *SERPINE-1* gene encoding PAI-1 protein by applying CRISPR/Cas9 genome editing techniques. To do so, we used lentiviral CRISPR/Cas9 Knockout (KO) and CRISPR/Cas9 Synergistic Activation Mediator (SAM) systems for *SERPINE-1* knockout and overexpression, respectively. SgRNAs selection and cloning as well as ESCs transduction procedures were performed according to the protocol precisely described in our recent study [25]. As displayed in Figure 6E and 6F, using the appropriate CRISPR/Cas9 system we were able to generate ESCs with SERPINE-1 knockout and overexpression, as indicated by RT-PCR and western blotting of genetically modified ESCs compared to ESCs used as transduction control (LV – containing sgRNA designed for SAM system but without Cas9).

To reveal the role of PAI-1 in SASP secreted by ESCs, we induced senescence in both control and genetically modified cells by applying sublethal oxidative treatment well described in our previous studies [18, 21, 22]. We then collected SASP from control and modified senescent ESCs and assessed levels of secreted PAI-1 using ELISA. As expected, we revealed the following distribution of PAI-1 content: senescent ESCs overexpressing *SERPINE-1* > senescent ESCs > senescent cells lacking functional *SERPINE-1* gene (Figure 6G). Using the above approach we were able to obtain 3 variants of SASP that remained specific to senescent ESCs, but differed in PAI-1 content.

Final set of experiments was focused on the estimation of the functional contribution of varied PAI-1 levels in SASP-induced senescence of young ESCs. To do so, young ESCs were cultured in CM obtained from senescent cells (LV) and genetically modified senescent cells. Notably, young cells cultured in CM from PAI-deficient senescent ESCs did not manifest any signs of paracrine senescence initiation, specifically their proliferation rate, cell size, autofluorescence and the activity of p53/p21/Rb pathway were similar to young cells (Figure 6H–6K). These findings suggest that PAI-1 may serve as the master-regulator of SASP-mediated senescence transduction within the population of young neighboring ESCs.

Summarizing all the above data, we can conclude that senescent ESCs are able to transduce senescence via SASP, thus adversely modifying their surroundings; PAI-1 secreted by senescent cells is probably the key SASP component responsible for senescence propagation in the population of ESCs.

## DISCUSSION

Normal functioning of ESCs that form stromal compartment of endometrial tissue seems to be crucial in terms of successful pregnancy outcomes. Firstly, during menstrual cycle ESCs undergo several stages, including active proliferation and tissue-specific differentiation [16, 17]. Both phases mediate maximal endometrial sensitivity, in other words receptivity, to invading embryo. Secondly, even before the direct attachment there is a so-called “secretome dialog” between the embryo and the maternal endometrium [26–29]. From the maternal side such a communication, at least in part, is provided by a tightly regulated secretory program of ESCs [26, 29]. In this context, changing the pattern of factors secreted by ESCs during senescence may have a great impact on the implantation process and, thus, on female fertility. Therefore, within the present study we focused predominantly on the investigation of the impact of senescent cells on young ESCs, as well as on the ascertainment of the precise combination of factors secreted by young and senescent ESCs, which to the best of our knowledge has not been yet investigated. Moreover, we were able to unravel the key molecular mediator of senescence propagation within ESCs population.

First of all, we tested what effect senescent ESCs may have on their normal, proliferation-prominent counterparts. As we revealed, co-culturing with senescent cells led to negative alterations in young ESCs functioning, namely decreased proliferation rate, increased lipofucine accumulation and cell hyperthrophy. Using 3D-coculturing scheme, we were able to obtain even more pronounced negative impact of senescent ESCs on young cells. To our knowledge, it is the first experimental evidence describing application of 3D-models to test effects of senescent cells on their young counterparts. Based on these data, we speculated that senescent ESCs may transmit damage to young cells at least in part via cell–cell contacts. In line with our observations, it was shown that senescent fibroblasts may induce DNA damage response and senescence in the neighboring cells via gap junctions [5]. Such a phenomenon was termed “bystander effect”. Later it was revealed that “bystander effect” is mediated by ROS and SASP produced by senescent cells [8].

Having established negative influence of senescent ESCs on young cells during direct co-culturing, we next addressed whether conditioned medium containing SASP could reproduce adverse effects of senescent cells. We revealed that conditioned medium from senescent ESCs had negative impact on the young cells. As expected, effects of CM-sen on the main properties of young ESCs were less pronounced compared to those upon co-culturing with senescent cells. Nevertheless, young ESCs cultured in CM-sen displayed most of the typical senescence markers, e.g. reduced proliferation, increased cell size, enhanced SA-β-Gal staining, activation of DDR and p53/p21 signaling cascade. Therefore, we concluded that that conditioned medium from senescent ESCs is sufficient to trigger senescence in young cells. Interestingly, the authors that discovered senescence transmitting via gap junctions were unable to obtain similar effects using conditioned medium from senescent cells [5]. However, the vast majority of studies performed using various cell types, including fibroblasts, MCF-7, MSCs, clearly demonstrate that conditioned medium containing SASP factors may mediate senescence progression within young cells populations [15, 30, 31]. The observed variations might be due to the differences in cell origin, senescence inducer or experimental conditions.

Besides soluble factors secreted by senescent cells, recently it has emerged that senescent cells also communicate by releasing EVs that can act on nearby cells [32]. Therefore, along with other secreted factors, EVs are now considered as the biologically active SASP components [6, 7, 32, 33]. Bearing in mind senescence-inducing properties of full SASP produced by senescent ESCs, we next checked what fraction – soluble one or EV – may primarily transmit senescence to young ESCs. The ability of EV produced by senescent cells to trigger senescence in young cells, similar to SASP, was previously described for endothelial cells [34]. Also, it was shown that EV obtained from the bone marrow interstitial fluid of aged mice accelerated senescence of bone marrow stem cells [35]. However, functional consequences of EV and SF are not always unidirectional. For example, EV secreted by senescent retinal pigment epithelial RPE-1 cells displayed pro-tumorigenic effect on MCF-7 cells similar to full SASP, whereas these effects were attenuated when using only SF [36]. According to our data, both SF and EV secreted by senescent ESCs turned out to be responsible for paracrine senescence propagation in the population of young cells, though the adverse influence of SF produced by senescent ESCs was more significant compared to EV. Together the above results indicate that senescent ESCs may transmit senescence within the cell population via cell contacts, secreted soluble factors and EV. To sum up, senescent ESCs appeared within endometrium lose their proliferation ability and may spread senescence on the neighboring normal cells, leading to tissue malfunction and reduced plasticity, thus forming kind of “vicious circle”.

We then focused on the precise alterations of the secretory profile during ESCs senescence, as it may impair endometrial receptivity to invading trophoblast [26–29]. By performing a secretome-wide profiling of young and senescent ESCs by LC-MS/MS, we have identified a subset of proteins characterizing their senescent phenotype, several of which are involved in extracellular matrix (ECM) remodeling, immune response, regulation of cell migration and positive regulation of apoptosis. Correct regulation of all the indicated processes is extremely important in the context of normal endometrial functioning and pregnancy progression. The endometrial extracellular matrix remodeling has a crucial role in the establishment of a successful pregnancy by playing a specific role in the trophoblast invasion, placentation, cell death and formation of the proper and functional implantation chamber around the embryo [37]. Furthermore, it was shown that prolonged secretion of pro-inflammatory factors by ESCs coincided with implantation failures during in vitro fertilization cycles [29]. Therefore, changes in ESCs secretome content caused by cell senescence may considerably impair the proper endometrial structure and sensitivity to embryo signaling.

Among a plenty of up-regulated proteins within the secretome of senescent ESCs compared to control ones, we paid our attention to the ECM-associated protein PAI-1. The level of PAI-1 secreted by control ESCs was rather high compared to other proteins secreted by ESCs. Moreover, protein content of PAI-1 rose significantly upon senescence induction. PAI-1 is a direct transcriptional target of p53 and the major inhibitor of fibrinolytic system [38]. Increase in PAI-1 expression accompanies both replicative senescence and stress-induced senescence [30, 38]. More recently, by applying LC-MS/MS enhanced secretion of PAI-1 was indicated for senescent adipose and bone marrow mesenchymal stromal cells [39]. Our results correlate well with the above data, as we revealed both enhanced expression and secretion of PAI-1 in senescent ESCs.

Today PAI-1 is considered not only as a marker but also as a key mediator of cellular senescence [40]. It was shown that PAI-1 deficient fibroblasts proliferate longer compared to wild-type ones, suggesting senescence resistance of the deficient cells [38]. Contrarily, overexpression of PAI-1 was sufficient to induce replicative senescence in fibroblasts [38]. Within the present study we were able to obtain both PAI-1 knockout and overexpressing ESCs by applying CRISPR/Cas9 genome editing techniques. Interestingly, we did not reveal any significant differences in proliferation rate between control and genetically modified cells (data not shown). However, by estimating effects of SASP from senescent PAI-knockout or overexpressing cells on the fate of young ESCs, we clearly demonstrated PAI-1 dependency in paracrine senescence induction. Namely, the more PAI-1 is secreted by senescent cells, the worse SASP affects young ESCs, whereas when PAI-1 is absent among the factors secreted by senescent ESCs, SASP has minimal if any negative influence on young cells. Based on these results, we can assume that PAI-1 secreted by senescent cells indeed can serve as a mediator of paracrine senescence within the population of ESCs. In line with this suggestion, it was shown that addition of purified recombinant PAI-1 protein to the culture medium of MCF-7 cells resulted in senescence induction [30]. Another confirmation was obtained by applying recombinant truncated form of PAI-1 (dominant negative) that blunted irradiation-induced pneumocyte senescence by competing against and decreasing the senescence-promoting actions of endogenous wild-type PAI-1 [41]. Though the above studies indicate senescence promoting role of PAI-1 protein, such conclusions were made based on the application of the exogenous protein. Here, by applying targeted genome editing techniques we were able to investigate the role of PAI-1 endogenously expressed and secreted by senescent cells. Therefore, our data is the first that performs a direct confirmation that PAI-1 produced by senescent cells mediates senescence propagation within cell population.

In terms of female fertility enhanced levels of PAI-1 secreted by senescent ESCs should be specifically highlighted. Being a key regulator of proteolysis and maternal tissue remodeling, PAI-1 has dual role during trophoblast invasion [42]. On the one hand, PAI-1 inhibits trophoblast invasion by inhibiting uPA; on the other hand, it may initiate or intensify the trophoblast invasion process [43, 44]. Prior to implantation the level of PAI-1 should be low, which is necessary for active proteolysis of the ECM components and for preparing the site for implantation. However, immediately after the implantation, the level of PAI-1 increases, since the maintenance of proteolytic activity may interfere with the development of an implanted embryo. Therefore, the optimal balance in the levels of secreted PAI-1 during implantation process controls accuracy and timeliness of trophoblast invasion and adhesion [42]. Thus, significantly increased levels of PAI-1 produced by senescent ESCs, may not only be responsible for triggering paracrine senescence in the neighboring cells, but also, may play role in the prevention of trophoblast invasion mediating various reproductive diseases. Indeed, it was shown that elevated levels of PAI-1 accompany such pathologies as recurrent pregnancy loss, repeated implantation failures and unexplained female infertility [42, 45, 46].

In conclusion, we revealed that PAI-1 secreted by senescent ESCs may serve as the master-regulator of paracrine senescence progression within the population of the neighboring ESCs. Based on these findings we can speculate that PAI-1 antagonists may provide a novel approach in preventing endometrial dysfunction and impaired embryo implantation. Further precise experimental studies on this topic can make contribution in the understanding of molecular basis of such infertility diseases as recurrent pregnancy losses and repeated implantation failures.

## MATERIALS AND METHODS

### Cell cultures

Human endometrial stromal cells (ESCs), previously isolated from desquamated endometrium in menstrual blood from healthy donor (line 2804), were cultured in DMEM/F12 (Gibco BRL, USA) supplemented with 10 % FBS (HyClone, USA), 1 % penicillin-streptomycin (Gibco BRL, USA) and 1 % glutamax (Gibco BRL, USA) at 37 °C in humidified incubator, containing 5 % CO_2_ [47]. Serial passaging was performed when the cells reached 80%–90% confluence. For the experiments, ESCs at early passages (between 8 and 12 passages) were seeded at a density of 7.5*10^3^ cells per cm^2^. HEK293T were cultured in DMEM (Biolot, Russian Federation)

The study was reviewed and approved by the Local Bioethics Committee of the Institute of Cytology Russian Academy of Sciences RAS, protocol #2. The copy of the approval by the Bioethics Committee of the Institute of Cytology is available upon request.

### Senescence induction procedure

The premature senescence in ESCs was induced by sublethal oxidative stress according to the previously designed scheme [18, 21, 22]. Cells at subconfluent density (15*10^3^ per cm^2^) were treated with H_2_O_2_ at the concentration 200 μM for 1 h. After that, cells were washed twice with PBS to remove H_2_O_2_ and then cultured in fresh complete medium for at least 7 days until reaching the irreversible senescence. H_2_O_2_ stock solution in serum-free medium was prepared from 30 % H_2_O_2_ (Sigma, USA) just before adding.

### Lentiviral transduction

Protocols of lentiviral particles production, design and cloning of sgRNAs coding sequences for *SERPINE1* gene targeting, and ESCs lentiviral transduction are described in detail in our previous article [25]. For ESCs labeling in co-culturing experiments the backbone pUltraHot plasmid coding mCherry reporter was used (a gift from Malcolm Moore, Addgene plasmid #24130). For CRISPR-mediated PAI-1 expression modulation lenti dCAS-VP64_Blast, lenti MS2-P65-HSF1_Hygro, lenti sgRNA(MS2)_zeo backbone and lentiCRISPR v2 plasmids were used (gifts from Feng Zhang, Addgene plasmids #61425, #61426, #61427 and #52961).

### Flow cytometry analysis

Measurements of proliferation, cell size, cell autofluorescence, and ROS were carried out by flow cytometry using the CytoFLEX (Beckman Coulter, USA). The obtained data were analyzed using CytExpert software version 1.2. Adherent cells were rinsed twice with PBS and harvested by trypsinization. Detached cells were pooled and resuspended in fresh medium and then counted and analyzed for autofluorescence. In order to assess cell viability, just before analysis 50 μg/mL propidium iodide (PI) was added to each sample and mixed gently. The cell size was evaluated by cytometric forward light scattering of PI-negative cells. For the measurement of intracellular ROS levels 2’, 7’-dichlorodihydrofluorescein diacetate (H_2_DCF-DA, Invitrogen, USA) was used, according to the manufacturer’s instructions. Cells were loaded with 10 μM H_2_DCF-DA in serum-free medium and incubated in the dark for 20 min at 37 ºC, then harvested by tripsinization and suspended in a fresh medium. Cell fluorescence was immediately analyzed by flow cytometry with the peak excitation wavelength for oxidized DCF 488 nm and emission 525 nm. At least 10^4^ cells were measured per sample.

### Spheroid formation

Spheroids were formed from ESCs using the hanging drop technique [48]. 7*10^3^ cells per 35 μL were placed in drops on the cover of 100 mm culture dishes and then inverted over the dish. Cells spontaneously aggregated in hanging drops for 48 h, then were transferred for 48 h in dishes coated with 2-hydroxyethyl methacrylate (HEMA; Sigma-Aldrich, USA). Single cell suspension was obtained by spheroid treatment with 0.05 % trypsin/EDTA and used to monitor cell properties.

### CM collection and concentration

ESCs were seeded at a density of 15*10^3^ cells per cm^2^ and cultured for 24 h in complete medium. Then the medium was replaced with fresh DMEM/F12 without FBS for 16 h. Culture supernatants were collected and centrifuged at 2 500 × *g* for 10 min at +4 °C to remove cell debris. The clarified supernatants were then concentrated with a 3 000-Da-cutoff Centriprep spin columns (Millipore, USA). The samples were further concentrated using a 3 000-Da-cutoff Microcon spin columns (Amicon, USA). For WB samples were supplemented with Proteinase inhibitor cocktail (Sigma-Aldrich, USA) and lysed with Gold Lysis Buffer (1 % Triton X-100, 30 mM Tris-HCl (pH 8.0), 137 mM Sodium chloride, 15 % Glycerol, 5 mM EDTA).

### Wound scratch test

The migration of ESCs was assessed by wound healing assay. Cells at 100 % confluence were scratched with a sterile 200 μL pipette tip, washed, and then incubated either in complete growth medium or CM supplemented with 10% FBS for the indicated time. The initial wounding and the migration of cells in the scratched area were observed and photographed under the inverted microscope over a time periods of 0, 8 and 24h.

### SA-β-Gal activity

Cells expressing senescence associated β-galactosidase were detected with the use of senescence β-galactosidase staining kit (Cell Signaling Technology, USA) according to the manufacturer’s instructions. The kit detects β-galactosidase activity at pH 6.0 in cultured cells which is present only in senescent cells and is not found in pre-senescent, quiescent or immortal cells. Quantitative analysis of images was performed as described previously [20]. For each experimental point not less than 100 randomly selected cells were analyzed.

### Western blotting

Western blotting was performed as described previously [22]. SDS-PAGE electrophoresis, transfer to nitrocellulose membrane and immunoblotting with ECL (Thermo Scientific, USA) detection were performed according to standard manufacturer’s protocols (Bio-Rad Laboratories, USA). Antibodies against the following proteins were used: glyceraldehyde-3-phosphate dehydrogenase (GAPDH) (clone 14C10) (1:1000, #2118, Cell Signaling, USA), phospho-p53 (Ser15) (clone 16G8) (1:700, #9286, Cell Signaling, USA), p21Waf1/Cip1 (clone 12D1) (1:1000, #2947, Cell Signaling, USA), phospho-Rb (Ser807/811) (1:1000, #8516, Cell Signaling, USA), phospho-ATM (Ser1981) (clone D6H9) (1:1000, #5883, Cell Signaling, MA, USA), phospho-Histone H2A.X (Ser139) (clone JBW301) (1:1000, #05-636, Merck Millipore, Germany), CD63 (1:500, ab68418, Abcam, UK), HSP70 (clone 2H9) (#MABE1130, Merck Millipore, Germany), PAI-1 (D9C4) (1:1000, #11907, Cell Signaling, USA) as well as horseradish peroxidase-conjugated goat anti-rabbit IG (GAR-HRP, Cell Signaling, USA) (1:10000) and antimouse IG (GAM-HRP, Cell Signaling, USA) (1:10000). Full size blots are provided in the Supplementary Figure 1.

### Isolation of soluble factors (SF) and extracellular vesicles (EV)

To isolate EV the CM was collected as described above. EV contained in the resulting supernatant were sedimented by ultracentrifugation at >100,000 × *g* for 2 h at +4 °C. The supernatants containing SF were collected and stored at +4 °C. The EV pellets were suspensed thoroughly in culture medium and then stored in glass at +4 °C to avoid sticking to plastic. The maximum time of EV and SF storage was limited by the duration of a separate experiment, but was no longer than 10 days. For Western blotting, EV pellets were immediately lysed with Low RIPA (20 mM Tris-HCl pH 7.5; 20 mM NaCl; 0.1 % Triton X-100; 0.1 mM EDTA; 0.2 mM PMSF), sonicated in ultrasonic bath for 1 min and then precipitated in absolute acetone overnight at −20 °C. The protein pellets were mixed with 1x Laemmli buffer, boiled at 99 °C and then used for analysis or stored at −20 °C. The SF were concentrated and prepared for WB as described above for CM.

### CM preparation for LC-MS/MS analysis

CM-ctr and CM-sen (100 mL) were clarified by centrifugation, lyophilized and resuspended in 5.6 mL of 50 mM NH_4_HCO_3_ pH 8.0. The proteins were then precipitated with 20 % trichloroacetic acid for 45 min on ice and centrifuged for 15 min at 17500 × *g*. Pellets washed with diethyl ether and acetone were air dried at room temperature, resuspended in 25 μL Laemmli buffer, mixed, pooled and heated at 95 °C for 2 min. Then proteins were separated onto a NuPAGE™ 4−12 % Bis-Tris Protein SDS-PAGE Gel (Invitrogen, CA, USA), and gel lanes were subsequently excised and chopped. Proteins were reduced in-gel with 2.5 mM dithiothreitol (final concentration) at 60 °C for 30 min and carbamidomethylated with 7.5 mM iodoacetamide (final concentration) at room temperature in the dark for 30 min. Enzymatic digestion was performed by the addition of 100 μL of Sequencing Grade Modified Trypsin (Promega, WI, USA) to each gel lane. Digestion was performed by incubation at 37 °C for 15 h. Extraction of tryptic peptides was firstly performed in 25 μL of 1 % TFA, and then in 25 μL of 0.1% TFA/50 %, at room temperature. Extracts were reduced to 5−10 μL, diluted twice with 35 μL of 0.1% TFA, and then dried under vacuum in a SpeedVac Concentrator (Savant Instruments, NY, USA). Samples were resuspended in H_2_O/CH_3_CN/formic acid (FA) 95 %/5 %/0,1 %, centrifuged at 17 500 x g for 10 min. Aliquots of the supernatant (6 μL) were analyzed in triplicate by LC-MS/MS.

### LC-MS/MS configuration and protein identification

Electrospray ionization (ESI) linear ion trap quadrupole (LTQ)-Orbitrap MS was performed on a LTQ Orbitrap Velos from Thermo Electron (San Jose, CA, USA) equipped with a NanoAcquity system from Waters (Waters Corporation, Manchester, UK). Peptides were trapped on a home-made 5 μm 200 Å Magic C18 AQ (Michrom, Auburn, CA, USA) 0.1 × 20 mm pre-column and separated on a home-made 5 μm 100 Å Magic C18 AQ (Michrom) 0.75 × 150 mm column with a gravity-pulled emitter. The analytical separation was run for 65 min, using a gradient of H_2_O/FA 99.9 %/0.1 % (solvent A) and CH_3_CN/FA 99.9 %/0.1 % (solvent B), at a flow rate of 220 nL/min as follows: 5 % solvent B for 1 min, from 5 to 35 % solvent B in 5 min and from 35 to 80 % solvent B in 10 min. For MS survey scans, the Orbitrap resolution was set to 60 000 and the ion population was set to 5 × 10^5^ with an m/z window from 400 to 2000. Maximum of 3 precursors were selected for both collision-induced dissociation (CID) in the LTQ and high-energy C-trap dissociation (HCD) with analysis in the Orbitrap. For MS/MS in the LTQ, the ion population was set to 7000 (isolation width of 2 m/z) while for MS/MS detection in the OT, it was set to 2 × 10^6^ (isolation width of 2.5 m/z), with resolution of 7500, first mass at m/z = 100, and maximum injection time of 750 ms. The normalized collision energies were set to 35 % for CID and 60 % for HCD.

The monoisotopic masses of the selected precursor ions were corrected using an in-house written Perl script [49]. The corrected mgf files, combined from the 8 analyzed gel lanes from each condition, were searched against the Uniprot_sprot database (release 2014_10) by using Mascot software (Matrix Science, London, UK; version 2.2.07). Homo Sapiens taxonomy (20 194 entries) was specified for database searching. The fragment and parent ion tolerance were set to 0.60 Da and 10 ppm, respectively. Trypsin was selected as the digestion enzyme, with one potential missed cleavage. Carbamidomethylation of cysteine and oxidation of methionine were selected as fixed and variable modifications, respectively. Scaffold software (version Scaffold_4.4.1.1, Proteome Software Inc., Portland, OR, USA) was used to analyze MS/MS-based peptide and protein identifications. Peptide identifications were accepted if they could be established at greater than 95.0 % probability by the Peptide Prophet algorithm with Scaffold delta-mass correction [50]. Protein identifications were accepted if they could be established at greater than 99.0 % probability and contained at least 2 identified peptides. Protein probabilities were assigned by the Protein Prophet algorithm [51]. Proteins that contained similar peptides and could not be differentiated based on MS/MS analysis alone were grouped to satisfy the principles of parsimony.

The mass spectrometry proteomics data have been deposited to the ProteomeXchange Consortium via the PRIDE [52] partner repository with the dataset identifier PXD015742 and 10.6019/PXD015742.

### Quantitative analysis and bioinformatics

To identify differentially expressed proteins, relative quantification was performed by applying a Scaffold label free approach based on total spectra quantification (i.e, the sum of all the spectra associated with a specific protein within a sample). Normalized spectral counts were calculated by dividing the spectral counts for an identified protein by the sum of the spectral counts per sample. The statistical evaluation of proteins differentially expressed between the CM-ctr and CM-sen samples was performed by applying the T-test with Hochberg-Benjamini correction (p<0.05).

Sets of up- and down-regulated secreted proteins were converted into lists of according genes and characterized by Functional Enrichment Analysis carried out in Biological processes Gene Ontology (GO) terms using the Cluster-profiler package in R software [53]. The Benjamini method was used to control the false discovery rate (FDR) to correct the p-value [53].

### ELISA

The amounts of secreted PAI-1 were measured in the concentrated conditioned media by the Human PAI-1 ELISA Kit (SERPINE1) (ab184863, Abcam, UK) according to manufacturer’s instructions. To determine the concentration of PAI-1 in samples, GraphPad Prism 5 was used.

### RNA extraction, reverse transcription and real time PCR

For RNA extraction 2*10^6^ ESCs were lysed by ExtractRNA reagent (Evrogen, Russia). Further purification of RNA was carried out by standard phenol-chloroform extraction. The integrity of the isolated RNA was checked using RNA gel electrophoresis by the absence of degradation of RNA bands corresponding to the 18S and 28S rRNA subunits. The purity and concentration of RNA samples was assessed spectrophotometrically using Thermo Scientific NanoDrop 2000.

Reverse transcription was performed using the MMLV RT kit (Evrogen, Russia), according to the manufacturer’s instructions. The reaction mixtures of 20 μl volume included: 1× buffer for the synthesis of the first chain, 0.5 mM dNTP, 2 mM of DTT, 1 μM of random decanucleotide primers, 1 u of MMLV and 1 μg of total RNA from samples. The reactions of cDNA synthesis were carried out as follows: 10 min +25 °C, 120 min +42 °C, 10 min +70 °C, reaction volume 20 μl, Tcap +40 °C.

Gene expression levels were assessed using the Real-time PCR BioRad CFX-96 amplifier (BioRad, USA). Primer sequences were developed using web applications Primer3web (version 4.1.0) and IDT OligoAnalyzer and listed in Table 1, GAPDH was used as the reference gene. The absence of non-specific amplification and dimer formation of primers was verified by setting negative controls without using the cDNA template and analyzing the melting curves of the amplification products. Reagents from the qPCRmix-HS SYBR Evrogen kit (Evrogen, Russia) were used for the reaction. The reaction mixtures of 20 μl volume included: 0.5 μM of forward and reverse primers, 1× reaction buffer and 3 μl of diluted cDNA samples. Reaction program: initial melting at +95 °C 5 min; 39 cycles of melting at +95 °C 10 sec, annealing at +62 °C 15 sec, and synthesis at + 72 °C 15 sec; melting in the temperature range from +65 °C to +95 °C in increments of 0.5 °C in 5 sec. Each experimental sample was set in three technical replicates. The analysis of the obtained data was performed using the Bio-Rad CFX Manager software (BioRad, USA).

**Table 1:** Primer oligonucleotide sequences.

**N**	**Oligonucleotide**	**Sequence**
1	PAI-1 forward	5′-CAGAAACAGTGTGCATGGGTTA-3′
2	PAI-1 reverse	5′-CACGCATCTGACATTTCTTCCT-3′
3	GAPDH forward	5′-GAGGTCAATGAAGGGGTCAT-3′
4	GAPDH reverse	5′-AGTCAACGGATTTGGTCGTA-3′

### Statistical analysis

Unless otherwise indicated, all quantitative data are shown as M ± S.D. To get significance in the difference between two groups two-sided t-test or Wilcoxon-Mann-Whitney rank sum test were applied. For multiple comparisons between groups, ANOVA with Tukey HSD was used. Statistical analysis was performed using R software [54].

## Supplementary Material

Supplementary Figure 1

Supplementary Table 1

Supplementary Table 2

Supplementary Table 3

Supplementary Table 4
