# Supplementary figures and images for: Molecular basis of senescence transmitting in the population of human endometrial stromal cells
Source: Aging (Albany NY). 2019 Nov 5;11(21):9912–31. doi: 10.18632/aging.102441 (PMC6874437; doi:10.18632/aging.102441)

SUPPLEMENTARY FIGURE

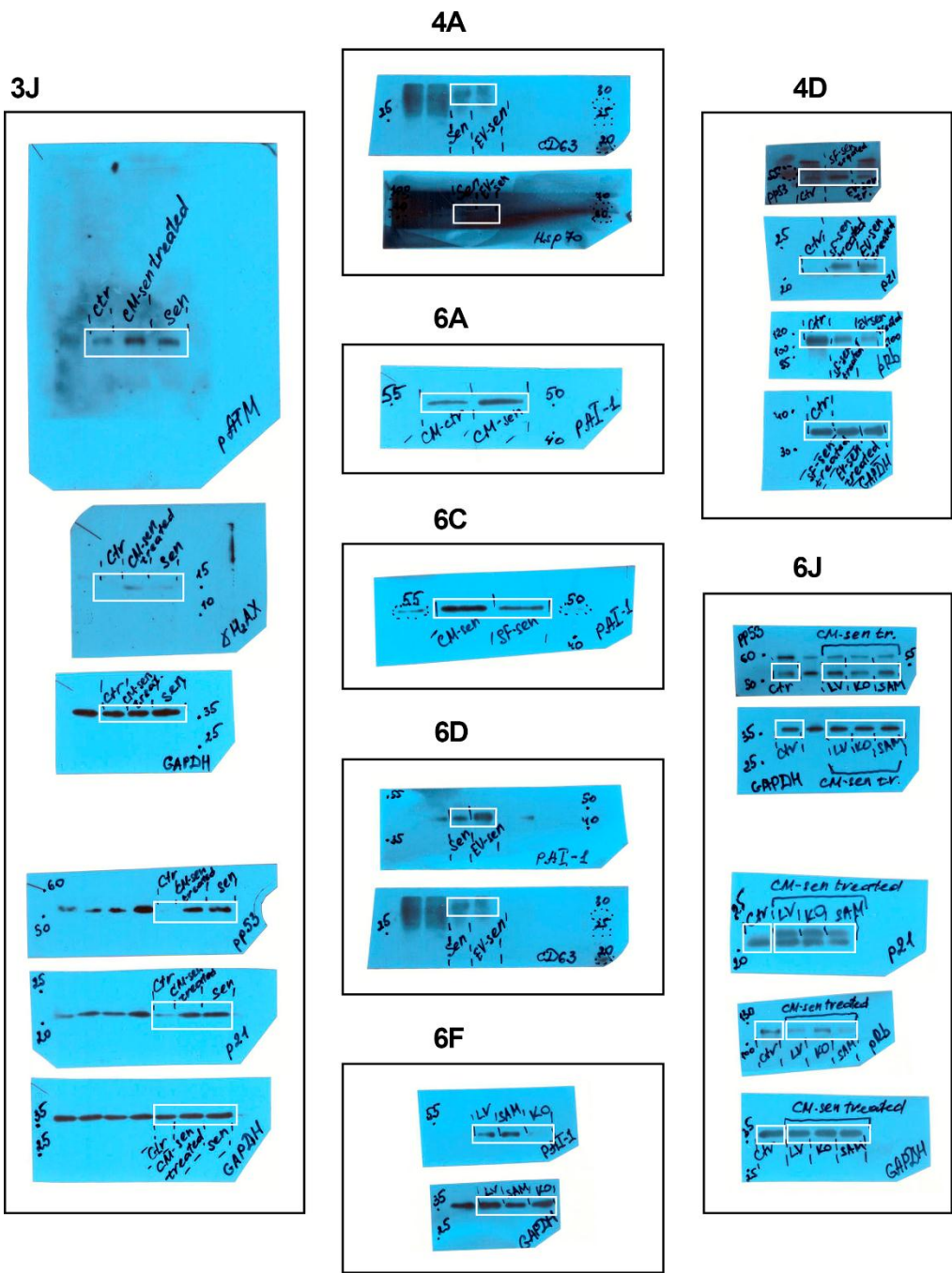

Supplement: Supplementary Figure 1 [file aging-11-102441-s003.pdf]
