# Supplementary material for: Molecular basis of senescence transmitting in the population of human endometrial stromal cells
Source: Aging (Albany NY). 2019 Nov 5;11(21):9912–31. doi: 10.18632/aging.102441 (PMC6874437; doi:10.18632/aging.102441)
Supplement: Supplementary Table 1 [file aging-11-102441-s002.pdf]

Supplementary Table 1. Proteins detected uniquely in CTR ESCs secretome

| Identified Proteins (141) | Uniprot ID | MW (kDa) | Condition (max # unique peptides) | |
|---|---|---|---|---|
| | | | Ctrl | Sen |
| Serotransferrin | TRFE_HUMAN | 77 | 34 | 0 |
| DNA damage-binding protein 1 | DDB1_HUMAN | 127 | 25 | 0 |
| Aminopeptidase B | AMPB_HUMAN | 73 | 15 | 0 |
| Thimet oligopeptidase | THOP1_HUMAN | 79 | 15 | 0 |
| Phosphoserine aminotransferase | SERC_HUMAN | 40 | 13 | 0 |
| DNA-(apurinic or apyrimidinic site) lyase | APEX1_HUMAN | 36 | 12 | 0 |
| Glutathione reductase, mitochondrial | GSHR_HUMAN | 56 | 12 | 0 |
| Transferrin receptor protein 1 | TFR1_HUMAN | 85 | 12 | 0 |
| Glucosamine-6-phosphate isomerase 2 | GNPI2_HUMAN | 31 | 11 | 0 |
| Endoplasmic reticulum aminopeptidase 1 | ERAP1_HUMAN | 107 | 10 | 0 |
| Glucosamine-6-phosphate isomerase 1 | GNPI1_HUMAN | 33 | 10 | 0 |
| Glycogen phosphorylase, liver form | PYGL_HUMAN | 97 | 10 | 0 |
| Guanine nucleotide-binding protein subunit beta-2-like 1 | GBLP_HUMAN | 35 | 10 | 0 |
| Membrane-bound transcription factor site-1 protease | MBTP1_HUMAN | 118 | 10 | 0 |
| Vacuolar protein sorting-associated protein 26A | VP26A_HUMAN | 38 | 10 | 0 |
| Adenosine kinase | ADK_HUMAN | 41 | 9 | 0 |
| Bifunctional purine biosynthesis protein PURH | PUR9_HUMAN | 65 | 9 | 0 |
| Ezrin | EZRI_HUMAN | 69 | 9 | 0 |
| Ig gamma-1 chain C region | IGHG1_HUMAN | 36 | 9 | 0 |
| Acylamino-acid-releasing enzyme | ACPH_HUMAN | 81 | 8 | 0 |
| Exostosin-2 | EXT2_HUMAN | 82 | 8 | 0 |
| Glutamate dehydrogenase 1, mitochondrial | DHE3_HUMAN | 61 | 8 | 0 |
| Proliferation-associated protein 2G4 | PA2G4_HUMAN | 44 | 8 | 0 |
| Alcohol dehydrogenase class-3 | ADHX_HUMAN | 40 | 7 | 0 |
| Aspartyl aminopeptidase | DNPEP_HUMAN | 52 | 7 | 0 |
| Coatomer subunit delta | COPD_HUMAN | 57 | 7 | 0 |
| Ig alpha-1 chain C region | IGHA1_HUMAN | 38 | 7 | 0 |
| Neurolysin, mitochondrial | NEUL_HUMAN | 81 | 7 | 0 |
| Phosphoribosylformylglycinamidine synthase | PUR4_HUMAN | 145 | 7 | 0 |
| Serpin B8 | SPB8_HUMAN | 43 | 7 | 0 |
| Alpha-mannosidase 2 | MA2A1_HUMAN | 131 | 6 | 0 |
| GTP-binding nuclear protein Ran | RAN_HUMAN | 24 | 6 | 0 |
| Heterogeneous nuclear ribonucleoprotein A1 | ROA1_HUMAN | 39 | 6 | 0 |
| Heterogeneous nuclear ribonucleoprotein A1-like 2 | RA1L2_HUMAN | 34 | 6 | 0 |
| Ig kappa chain C region | IGKC_HUMAN | 12 | 6 | 0 |
| Isopentenyl-diphosphate Delta-isomerase 1 | IDI1_HUMAN | 26 | 6 | 0 |
| Leukotriene A-4 hydrolase | LKHA4_HUMAN | 69 | 6 | 0 |
| Phosphoacetylglucosamine mutase | AGM1_HUMAN | 60 | 6 | 0 |
| Selenium-binding protein 1 | SBP1_HUMAN | 52 | 6 | 0 |
| Semaphorin-3A | SEM3A_HUMAN | 89 | 6 | 0 |
| Succinyl-CoA:3-ketoacid coenzyme A transferase 1, mitochondrial | SCOT1_HUMAN | 56 | 6 | 0 |
| Threonine--tRNA ligase, cytoplasmic | SYTC_HUMAN | 83 | 6 | 0 |
| 60 kDa SS-A/Ro ribonucleoprotein | RO60_HUMAN | 61 | 5 | 0 |
| Deoxyuridine 5'-triphosphate nucleotidohydrolase, mitochondrial | DUT_HUMAN | 27 | 5 | 0 |
| Fatty acid-binding protein, adipocyte | FABP4_HUMAN | 15 | 5 | 0 |
| Fibroblast growth factor 7 | FGF7_HUMAN | 23 | 5 | 0 |
| HLA class I histocompatibility antigen, A-69 alpha chain | 1A69_HUMAN | 41 | 5 | 0 |
| Lipocalin-1 | LCN1_HUMAN | 19 | 5 | 0 |
| NADH-cytochrome b5 reductase 2 | NB5R2_HUMAN | 31 | 5 | 0 |
| Proteasome subunit beta type-5 | PSB5_HUMAN | 28 | 5 | 0 |
| Ras-related protein Rap-1b | RAP1B_HUMAN | 21 | 5 | 0 |
| SPRY domain-containing protein 4 | SPRY4_HUMAN | 23 | 5 | 0 |

| | | | | |
|---|---|---|---|---|
| Thioredoxin-like protein 1 | TXNL1_HUMAN | 32 | 5 | 0 |
| Alpha-1-antitrypsin | A1AT_HUMAN | 47 | 4 | 0 |
| ATP-dependent RNA helicase DDX1 | DDX1_HUMAN | 82 | 4 | 0 |
| Coatomer subunit gamma-1 | COPG1_HUMAN | 98 | 4 | 0 |
| Eukaryotic translation initiation factor 6 | IF6_HUMAN | 27 | 4 | 0 |
| Glycogen phosphorylase, brain form | PYGB_HUMAN | 97 | 4 | 0 |
| Ig lambda-2 chain C regions | LAC2_HUMAN | 11 | 4 | 0 |
| Isochorismatase domain-containing protein 1 | ISOC1_HUMAN | 32 | 4 | 0 |
| Laminin subunit alpha-1 | LAMA1_HUMAN | 337 | 4 | 0 |
| Low-density lipoprotein receptor-related protein 6 | LRP6_HUMAN | 180 | 4 | 0 |
| Neutrophil gelatinase-associated lipocalin | NGAL_HUMAN | 23 | 4 | 0 |
| PITH domain-containing protein 1 | PITH1_HUMAN | 24 | 4 | 0 |
| Polypeptide N-acetylgalactosaminyltransferase 2 | GALT2_HUMAN | 65 | 4 | 0 |
| Putative deoxyribonuclease TATDN1 | TATD1_HUMAN | 34 | 4 | 0 |
| Putative hydrolase RBBP9 | RBBP9_HUMAN | 21 | 4 | 0 |
| Serpin B9 | SPB9_HUMAN | 42 | 4 | 0 |
| Xaa-Pro aminopeptidase 1 | XPP1_HUMAN | 70 | 4 | 0 |
| 3-ketoacyl-CoA thiolase, mitochondrial | THIM_HUMAN | 42 | 3 | 0 |
| Complement C3 | CO3_HUMAN | 187 | 3 | 0 |
| Desmocollin-3 | DSC3_HUMAN | 100 | 3 | 0 |
| Eukaryotic initiation factor 4A-I | IF4A1_HUMAN | 46 | 3 | 0 |
| Exostosin-1 | EXT1_HUMAN | 86 | 3 | 0 |
| Fumarylacetoacetate hydrolase domain-containing protein 2A | FAH2A_HUMAN | 35 | 3 | 0 |
| Hemicentin-1 | HMCN1_HUMAN | 613 | 3 | 0 |
| Hepatocyte growth factor-like protein | HGFL_HUMAN | 80 | 3 | 0 |
| Histone-binding protein RBBP4 | RBBP4_HUMAN | 48 | 3 | 0 |
| HLA class I histocompatibility antigen, A-2 alpha chain | 1A02_HUMAN | 41 | 3 | 0 |
| HLA class I histocompatibility antigen, Cw-12 alpha chain | 1C12_HUMAN | 41 | 3 | 0 |
| Ig gamma-2 chain C region | IGHG2_HUMAN | 36 | 3 | 0 |
| Kynurenine--oxoglutarate transaminase 3 | KAT3_HUMAN | 51 | 3 | 0 |
| LanC-like protein 1 | LANC1_HUMAN | 45 | 3 | 0 |
| Matrilin-2 | MATN2_HUMAN | 107 | 3 | 0 |
| Mycophenolic acid acyl-glucuronide esterase, mitochondrial | ABHDA_HUMAN | 34 | 3 | 0 |
| Myeloperoxidase | PERM_HUMAN | 84 | 3 | 0 |
| Polymeric immunoglobulin receptor | PIGR_HUMAN | 83 | 3 | 0 |
| Probable aminopeptidase NPEPL1 | PEPL1_HUMAN | 56 | 3 | 0 |
| Proliferating cell nuclear antigen | PCNA_HUMAN | 29 | 3 | 0 |
| Protein S100-A7 | S10A7_HUMAN | 11 | 3 | 0 |
| Protein S100-A9 | S10A9_HUMAN | 13 | 3 | 0 |
| Quinone oxidoreductase | QOR_HUMAN | 35 | 3 | 0 |
| Retinal rod rhodopsin-sensitive cGMP 3',5'-cyclic phosphodiesterase subunit delta | PDE6D_HUMAN | 17 | 3 | 0 |
| Rho GDP-dissociation inhibitor 2 | GDIR2_HUMAN | 23 | 3 | 0 |
| Sialic acid synthase | SIAS_HUMAN | 40 | 3 | 0 |
| Transthyretin | TTHY_HUMAN | 16 | 3 | 0 |
| Trifunctional purine biosynthetic protein adenosine-3 | PUR2_HUMAN | 108 | 3 | 0 |
| UDP-N-acetylhexosamine pyrophosphorylase | UAP1_HUMAN | 59 | 3 | 0 |
| Vacuolar protein sorting-associated protein 29 | VPS29_HUMAN | 21 | 3 | 0 |
| Zinc-alpha-2-glycoprotein | ZA2G_HUMAN | 34 | 3 | 0 |
| Aconitate hydratase, mitochondrial | ACON_HUMAN | 85 | 2 | 0 |
| Aldose 1-epimerase | GALM_HUMAN | 38 | 2 | 0 |
| Azurocidin | CAP7_HUMAN | 27 | 2 | 0 |
| Biliverdin reductase A | BIEA_HUMAN | 33 | 2 | 0 |
| Branched-chain-amino-acid aminotransferase, cytosolic | BCAT1_HUMAN | 43 | 2 | 0 |
| Branched-chain-amino-acid aminotransferase, mitochondrial | BCAT2_HUMAN | 44 | 2 | 0 |
| BRO1 domain-containing protein BROX | BROX_HUMAN | 46 | 2 | 0 |
| Clathrin heavy chain 1 | CLH1_HUMAN | 192 | 2 | 0 |
| Coatomer subunit beta' | COPB2_HUMAN | 102 | 2 | 0 |
| Complement C1q tumor necrosis factor-related protein 5 | C1QT5_HUMAN | 25 | 2 | 0 |
| Cytosolic non-specific dipeptidase | CNDP2_HUMAN | 53 | 2 | 0 |

| | | | | |
|---|---|---|---|---|
| Delta(3,5)-Delta(2,4)-dienoyl-CoA isomerase, mitochondrial | ECH1_HUMAN | 36 | 2 | 0 |
| Diphosphoinositol polyphosphate phosphohydrolase 1 | NUDT3_HUMAN | 19 | 2 | 0 |
| Four and a half LIM domains protein 1 | FHL1_HUMAN | 36 | 2 | 0 |
| Galectin-8 | LEG8_HUMAN | 36 | 2 | 0 |
| Glutathione S-transferase Mu 1 | GSTM1_HUMAN | 26 | 2 | 0 |
| Golgi resident protein GCP60 | GCP60_HUMAN | 61 | 2 | 0 |
| Guanine nucleotide-binding protein G(I)/G(S)/G(T) subunit beta-1 | GBB1_HUMAN | 37 | 2 | 0 |
| Haptoglobin | HPT_HUMAN | 45 | 2 | 0 |
| HLA class I histocompatibility antigen, A-24 alpha chain | 1A24_HUMAN | 41 | 2 | 0 |
| Hydroxyacyl-coenzyme A dehydrogenase, mitochondrial | HCDH_HUMAN | 34 | 2 | 0 |
| Ig gamma-4 chain C region | IGHG4_HUMAN | 36 | 2 | 0 |
| Inositol-3-phosphate synthase 1 | INO1_HUMAN | 61 | 2 | 0 |
| Kynurenine--oxoglutarate transaminase 1 | KAT1_HUMAN | 48 | 2 | 0 |
| Latexin | LXN_HUMAN | 26 | 2 | 0 |
| Malectin | MLEC_HUMAN | 32 | 2 | 0 |
| Neurotrophin-3 | NTF3_HUMAN | 29 | 2 | 0 |
| Neutral alpha-glucosidase AB | GANAB_HUMAN | 107 | 2 | 0 |
| Peroxiredoxin-2 | PRDX2_HUMAN | 22 | 2 | 0 |
| Phospholipid hydroperoxide glutathione peroxidase, mitochondrial | GPX4_HUMAN | 22 | 2 | 0 |
| Proteasome subunit beta type-7 | PSB7_HUMAN | 30 | 2 | 0 |
| Protein NipSnap homolog 3A | NPS3A_HUMAN | 28 | 2 | 0 |
| Protocadherin Fat 1 | FAT1_HUMAN | 506 | 2 | 0 |
| Secernin-2 | SCRN2_HUMAN | 47 | 2 | 0 |
| Serpin B4 | SPB4_HUMAN | 45 | 2 | 0 |
| Sorbitol dehydrogenase | DHSO_HUMAN | 38 | 2 | 0 |
| Stromal cell-derived factor 2 | SDF2_HUMAN | 23 | 2 | 0 |
| Stromal cell-derived factor 2-like protein 1 | SDF2L_HUMAN | 24 | 2 | 0 |
| Tetranectin | TETN_HUMAN | 23 | 2 | 0 |
| Transmembrane protein 132A | T132A_HUMAN | 110 | 2 | 0 |
| WD repeat-containing protein 5 | WDR5_HUMAN | 37 | 2 | 0 |
