# Supplementary material for: Molecular basis of senescence transmitting in the population of human endometrial stromal cells
Source: Aging (Albany NY). 2019 Nov 5;11(21):9912–31. doi: 10.18632/aging.102441 (PMC6874437; doi:10.18632/aging.102441)
Supplement: Supplementary Table 2 [file aging-11-102441-s004.pdf]

Supplementary Table 2. Proteins detected uniquely in SEN ESCs secretome

Identified Proteins (92)	Uniprot ID	MW (kDa)	Condition (max # unique peptides)	
			Ctrl	Sen
Multiple epidermal growth factor-like domains protein 8	MEGF8_HUMAN	303	0	10
Alpha-1,6-mannosylglycoprotein 6-beta-N-acetylglucosaminyltransferase A	MGT5A_HUMAN	85	0	9
3-hydroxyisobutyrate dehydrogenase, mitochondrial	3HIDH_HUMAN	35	0	9
Amphoterin-induced protein 2	AMGO2_HUMAN	58	0	7
Secreted frizzled-related protein 4	SFRP4_HUMAN	40	0	6
Peroxiredoxin-5, mitochondrial	PRDX5_HUMAN	22	0	6
Ras GTPase-activating-like protein IQGAP1	IQGA1_HUMAN	189	0	6
Myosin regulatory light polypeptide 9	MYL9_HUMAN	20	0	6
Leucine-rich repeat transmembrane protein FLRT2	FLRT2_HUMAN	74	0	6
PDZ and LIM domain protein 4	PDLI4_HUMAN	35	0	5
Importin-5	IPO5_HUMAN	124	0	5
Translin-associated protein X	TSNAX_HUMAN	33	0	5
Tubulin beta chain	TBB5_HUMAN	50	0	5
Serine/threonine-protein phosphatase 2A 65 kDa regulatory subunit A alpha isoform	2AAA_HUMAN	65	0	5
Adenylate kinase isoenzyme 1	KAD1_HUMAN	22	0	5
Proteasome activator complex subunit 2	PSME2_HUMAN	27	0	5
Glucosidase 2 subunit beta	GLU2B_HUMAN	59	0	5
Angiotensinogen	ANGT_HUMAN	53	0	4
Dynein light chain 1, cytoplasmic	DYL1_HUMAN	10	0	4
Ubiquitin-conjugating enzyme E2 L3	UB2L3_HUMAN	18	0	4
Calponin-1	CNN1_HUMAN	33	0	4
Peptidyl-prolyl cis-trans isomerase FKBP7	FKBP7_HUMAN	30	0	4
Retinol-binding protein 1	RET1_HUMAN	16	0	4
Intercellular adhesion molecule 5	ICAM5_HUMAN	97	0	4
Basal cell adhesion molecule	BCAM_HUMAN	67	0	4
Glypican-4	GPC4_HUMAN	62	0	4
Copper transport protein ATOX1	ATOX1_HUMAN	7	0	3
Cullin-associated NEDD8-dissociated protein 1	CAND1_HUMAN	136	0	3
Stromelysin-3	MMP11_HUMAN	55	0	3
CD81 antigen	CD81_HUMAN	26	0	3
Histidine triad nucleotide-binding protein 1	HINT1_HUMAN	14	0	3
Heat shock 70 kDa protein 13	HSP13_HUMAN	52	0	3
Tubulin-specific chaperone A	TBCA_HUMAN	13	0	3
Serine/threonine-protein phosphatase 2A activator	PTPA_HUMAN	41	0	3
Rho GTPase-activating protein 1	RHG01_HUMAN	50	0	3
Podocan	PODN_HUMAN	69	0	3

Latent-transforming growth factor beta-binding protein 3	LTBP3_HUMAN	139	0	3
Polyadenylate-binding protein 1	PABP1_HUMAN	71	0	3
Src substrate cortactin	SRC8_HUMAN	62	0	3
Sorting nexin-12	SNX12_HUMAN	20	0	3
Golgi membrane protein 1	GOLM1_HUMAN	45	0	3
Programmed cell death 1 ligand 2	PD1L2_HUMAN	31	0	3
Transcriptional activator protein Pur-alpha	PURA_HUMAN	35	0	3
Hornerin	HORN_HUMAN	282	0	3
Deoxycytidylate deaminase	DCTD_HUMAN	20	0	3
Palladin	PALLD_HUMAN	151	0	3
Insulin-like growth factor-binding protein-like 1	IBPL1_HUMAN	29	0	3
Alpha-L-iduronidase	IDUA_HUMAN	73	0	3
Synaptic vesicle membrane protein VAT-1 homolog	VAT1_HUMAN	42	0	2
Carboxypeptidase E	CBPE_HUMAN	53	0	2
Lysosomal acid phosphatase	PPAL_HUMAN	48	0	2
Inter-alpha-trypsin inhibitor heavy chain H2	ITIH2_HUMAN	106	0	2
Polymerase I and transcript release factor	PTRF_HUMAN	43	0	2
Niban-like protein 1	NIBL1_HUMAN	84	0	2
Protein phosphatase 1 regulatory subunit 7	PP1R7_HUMAN	42	0	2
Ubiquitin-fold modifier 1	UFM1_HUMAN	9	0	2
Layilin	LAYN_HUMAN	43	0	2
Protocadherin Fat 4	FAT4_HUMAN	543	0	2
SLIT and NTRK-like protein 4	SLIK4_HUMAN	94	0	2
Amyloid-like protein 2	APLP2_HUMAN	87	0	2
Phospholipase D3	PLD3_HUMAN	55	0	2
Isoamyl acetate-hydrolyzing esterase 1 homolog	IAH1_HUMAN	28	0	2
Syntenin-1	SDCB1_HUMAN	32	0	2
Heme-binding protein 1	HEBP1_HUMAN	21	0	2
Matrix Gla protein	MGP_HUMAN	12	0	2
Sulfatase-modifying factor 1	SUMF1_HUMAN	41	0	2
Leucine-rich repeat-containing protein 59	LRC59_HUMAN	35	0	2
Tissue factor pathway inhibitor	TFPI1_HUMAN	35	0	2
N-acetyllactosaminide beta-1,3-N-acetylglucosaminyltransferase	B3GN1_HUMAN	47	0	2
Calponin-3	CNN3_HUMAN	36	0	2
Cell adhesion molecule 1	CADM1_HUMAN	49	0	2
Acid ceramidase	ASAH1_HUMAN	45	0	2
Protein FAM198B	F198B_HUMAN	58	0	2
PDZ and LIM domain protein 2	PDLI2_HUMAN	37	0	2
Nucleolin	NUCL_HUMAN	77	0	2
Transcription elongation factor B polypeptide 1	ELOC_HUMAN	12	0	2
Apoptosis regulator BAX	BAX_HUMAN	21	0	2
Ras GTPase-activating protein-binding protein 1	G3BP1_HUMAN	52	0	2
A disintegrin and metalloproteinase with thrombospondin motifs 5	ATS5_HUMAN	102	0	2
HD domain-containing protein 2	HDDC2_HUMAN	23	0	2
Tight junction protein ZO-1	ZO1_HUMAN	195	0	2

Apolipoprotein E	APOE_HUMAN	36	0	2
Hypoxia up-regulated protein 1	HYOU1_HUMAN	111	0	2
Magnesium-dependent phosphatase 1	MGDP1_HUMAN	20	0	2
Cadherin-13	CAD13_HUMAN	78	0	2
Growth/differentiation factor 15	GDF15_HUMAN	34	0	2
Actin-related protein 2/3 complex subunit 5-like protein	ARP5L_HUMAN	17	0	2
Calcium-binding protein 39	CAB39_HUMAN	40	0	2
Apoptosis-associated speck-like protein containing a CARD	ASC_HUMAN	22	0	2
Olfactomedin-like protein 2A	OLM2A_HUMAN	73	0	2
Dynactin subunit 2	DCTN2_HUMAN	44	0	2
Alpha-N-acetylgalactosaminide alpha-2,6-sialyltransferase 5	SIA7E_HUMAN	38	0	2
