# Supplementary material for: Molecular basis of senescence transmitting in the population of human endometrial stromal cells
Source: Aging (Albany NY). 2019 Nov 5;11(21):9912–31. doi: 10.18632/aging.102441 (PMC6874437; doi:10.18632/aging.102441)
Supplement: Supplementary Table 3 [file aging-11-102441-s001.pdf]

Supplementary Table 3. Proteins up-regulated in SEN ESCs secretome, quantified with at least 2 spectral counts (sc)

Identified Proteins	Uniprot ID	MW (kDa)	Condition (max # sc/peptides)		Ratio Sen/Ctrl	*p-value*
			Ctrl	Sen		
Semaphorin-5A	SEM5A_HUMAN	121	2/2	23/15	16.63	< 0.00010
C-type mannose receptor 2	MRC2_HUMAN	167	7/3	80/26	13.29	< 0.00010
Matrix-remodeling-associated protein 5	MXRA5_HUMAN	312	16/9	158/47	10.43	< 0.00010
Glia-derived nexin	GDN_HUMAN	44	17/9	125/19	7.86	< 0.00010
Amyloid beta A4 protein	A4_HUMAN	87	5/5	32/12	6.75	< 0.00010
Testican-1	TICN1_HUMAN	49	6/5	33/16	6.34	< 0.00010
EMILIN-1	EMIL1_HUMAN	107	12/7	68/19	5.98	< 0.00010
Tropomyosin alpha-1 chain	TPM1_HUMAN	33	6/3	33/14	5.96	< 0.00010
Plexin domain-containing protein 2	PXDC2_HUMAN	60	2/2	12/7	5.60	< 0.00010
Receptor-type tyrosine-protein phosphatase gamma	PTPRG_HUMAN	162	3/3	17/7	5.39	< 0.00010
Adipocyte enhancer-binding protein 1	AEBP1_HUMAN	131	9/6	42/19	5.18	< 0.00010
Proteasome subunit alpha type-1	PSA1_HUMAN	30	3/2	14/9	5.13	< 0.00010
Plastin-3	PLST_HUMAN	71	28/21	127/35	4.99	< 0.00010
Flavin reductase (NADPH)	BLVRB_HUMAN	22	2/2	9/5	4.87	< 0.00010
CD109 antigen	CD109_HUMAN	162	16/10	67/29	4.39	< 0.00010
60 kDa heat shock protein, mitochondrial	CH60_HUMAN	61	8/6	27/18	3.55	< 0.00010
Pregnancy-specific beta-1-glycoprotein 4	PSG4_HUMAN	47	22/13	76/17	3.45	< 0.00010
Complement C1r subcomponent	C1R_HUMAN	80	36/20	103/24	3.33	< 0.00010
Profilin-1	PROF1_HUMAN	15	14/7	45/8	3.30	< 0.00010
Vinculin	VINC_HUMAN	124	39/20	124/45	3.25	< 0.00010
Chitinase-3-like protein 1	CH3L1_HUMAN	43	38/16	112/20	3.07	< 0.00010
14-3-3 protein zeta/delta	1433Z_HUMAN	28	17/6	51/16	2.94	< 0.00010
Tropomyosin alpha-4 chain	TPM4_HUMAN	29	11/7	30/9	2.86	< 0.00010
Collagen alpha-2(VI) chain	CO6A2_HUMAN	109	43/15	104/24	2.54	< 0.00010
Cathepsin D	CATD_HUMAN	45	12/7	28/11	2.48	< 0.00010
Latent-transforming growth factor beta-binding protein 2	LTBP2_HUMAN	195	52/24	121/39	2.37	< 0.00010

Plasminogen activator inhibitor 1	PAI1_HUMAN	45	109/21	243/23	2.26	< 0.00010
Transgelin	TAGL_HUMAN	23	34/15	74/16	2.20	< 0.00010
Stanniocalcin-2	STC2_HUMAN	33	14/4	30/4	2.15	< 0.00010
14-3-3 protein beta/alpha	1433B_HUMAN	28	12/6	25/11	2.15	< 0.00010
Carboxypeptidase Q	CBPQ_HUMAN	52	8/8	17/11	2.11	< 0.00010
Complement C1s subcomponent	C1S_HUMAN	77	36/18	72/19	2.01	< 0.00010
Vimentin	VIME_HUMAN	54	53/24	158/36	2.98	0.00010
Transforming growth factor-beta-induced protein ig-h3	BGH3_HUMAN	75	41/21	93/21	2.34	0.00011
Integrin alpha-V	ITAV_HUMAN	116	5/3	32/19	9.54	0.00012
Soluble scavenger receptor cysteine-rich domain-containing protein SSC5D	SRCRL_HUMAN	166	13/10	35/19	3.45	0.00013
Insulin-like growth factor-binding protein 3	IBP3_HUMAN	32	6/6	13/9	2.28	0.00014
Prostaglandin-H2 D-isomerase	PTGDS_HUMAN	21	17/3	41/7	2.54	0.00016
Inhibin beta A chain	INHBA_HUMAN	47	13/4	27/14	2.43	0.00016
Anthrax toxin receptor 1	ANTR1_HUMAN	63	3/3	24/11	7.79	0.00018
Lysosomal protective protein	PPGB_HUMAN	54	3/3	12/6	5.74	0.00019
Agrin	AGRIN_HUMAN	217	21/15	51/35	2.45	0.00022
Nucleoside diphosphate kinase B	NDKB_HUMAN	17	12/6	33/10	2.86	0.00022
Immunoglobulin superfamily containing leucine-rich repeat protein	ISLR_HUMAN	46	4/4	15/7	5.14	0.00024
Collagen alpha-1(XVIII) chain	COIA1_HUMAN	178	16/8	33/12	2.25	0.00026
Putative phospholipase B-like 2	PLBL2_HUMAN	65	10/6	29/10	3.02	0.00026
Arylsulfatase A	ARSA_HUMAN	54	3/3	13/7	4.55	0.00029
Transgelin-2	TAGL2_HUMAN	22	5/4	15/10	3.08	0.00031
Procollagen-lysine,2-oxoglutarate 5-dioxygenase 2	PLOD2_HUMAN	85	17/12	37/16	2.40	0.00032
Alpha-N-acetylglucosaminidase	ANAG_HUMAN	82	13/10	43/18	3.81	0.00032
Deoxyribonuclease-2-alpha	DNS2A_HUMAN	40	4/3	9/4	2.37	0.00032
Spondin-2	SPON2_HUMAN	36	32/10	121/14	3.48	0.00035
Inactive serine protease PAMR1	PAMR1_HUMAN	80	6/6	14/7	2.39	0.00036
14-3-3 protein gamma	1433G_HUMAN	28	10/7	30/11	2.95	0.00038
Periostin	POSTN_HUMAN	93	18/13	53/24	2.96	0.00039
Nidogen-2	NID2_HUMAN	151	10/7	36/22	4.26	0.00039
N-sulphoglucosamine sulphohydrolase	SPHM_HUMAN	57	4/4	14/10	3.04	0.00039
Beta-hexosaminidase subunit alpha	HEXA_HUMAN	61	28/14	59/19	2.03	0.00041

Insulin-like growth factor-binding protein 6	IBP6_HUMAN	25	13/5	41/7	3.05	0.00049
Poly(ADP-ribose) glycohydrolase ARH3	ARHL2_HUMAN	39	6/5	16/9	2.48	0.00050
Low-density lipoprotein receptor	LDLR_HUMAN	95	19/10	43/13	2.09	0.00054
Gremlin-1	GREM1_HUMAN	21	22/8	74/10	3.20	0.00058
Legumain	LGMN_HUMAN	49	7/5	16/11	2.85	0.00058
Integrin beta-1	ITB1_HUMAN	88	11/9	30/15	2.86	0.00061
Putative RNA-binding protein 3	RBM3_HUMAN	17	2/2	5/3	2.17	0.00062
Myosin-9	MYH9_HUMAN	227	4/3	26/13	8.09	0.00065
Myristoylated alanine-rich C-kinase substrate	MARCS_HUMAN	32	2/1	10/5	6.28	0.00076
Tropomyosin alpha-3 chain	TPM3_HUMAN	33	4/1	16/3	10.94	0.00079
Iduronate 2-sulfatase	IDS_HUMAN	62	2/2	15/8	12.56	0.00083
Lysosomal alpha-mannosidase	MA2B1_HUMAN	114	5/4	32/16	7.17	0.00084
Coiled-coil domain-containing protein 80	CCD80_HUMAN	108	2/1	10/8	5.08	0.00084
Platelet-derived growth factor C	PDGFC_HUMAN	39	7/6	20/10	2.85	0.00089
Prosaposin	SAP_HUMAN	58	2/2	27/13	10.92	0.00094
Follistatin-related protein 3	FSTL3_HUMAN	28	4/3	9/7	2.29	0.00097
14-3-3 protein eta	1433F_HUMAN	28	7/3	14/4	2.18	0.00098
Plexin-B2	PLXB2_HUMAN	205	4/4	10/7	2.54	0.0010
Complement decay-accelerating factor	DAF_HUMAN	41	5/5	23/7	5.73	0.0011
Group XV phospholipase A2	PAG15_HUMAN	47	4/3	14/8	4.12	0.0011
Lysosomal Pro-X carboxypeptidase	PCP_HUMAN	56	11/6	29/9	2.61	0.0012
N-acetylglucosamine-6-sulfatase	GNS_HUMAN	62	22/11	41/13	2.10	0.0014
14-3-3 protein theta	1433T_HUMAN	28	9/3	31/11	3.70	0.0014
Myosin light chain kinase, smooth muscle	MYLK_HUMAN	211	2/1	7/4	2.79	0.0014
Extracellular superoxide dismutase [Cu-Zn]	SODE_HUMAN	26	22/8	55/10	2.75	0.0015
Pregnancy-specific beta-1-glycoprotein 5	PSG5_HUMAN	38	10/3	29/1	3.04	0.0015
Caldesmon	CALD1_HUMAN	93	8/6	40/16	6.41	0.0016
Protein S100-A11	S10AB_HUMAN	12	3/3	6/4	2.27	0.0016
Protein NOV homolog	NOV_HUMAN	39	5/3	9/5	2.38	0.0018
Brain acid soluble protein 1	BASP1_HUMAN	23	3/2	8/4	3.40	0.0018
Thrombospondin-3	TSP3_HUMAN	104	2/2	18/8	7.79	0.0018
Serine protease 23	PRS23_HUMAN	43	4/3	9/5	2.29	0.0019
Calmodulin	CALM_HUMAN	17	2/1	12/7	14.58	0.0019
14-3-3 protein epsilon	1433E_HUMAN	29	22/13	40/15	2.11	0.0020
Dipeptidyl peptidase 2	DPP2_HUMAN	54	5/5	13/10	3.06	0.0021
Proteasome subunit alpha type-2	PSA2_HUMAN	26	6/3	12/8	2.57	0.0025

Reticulocalbin-1	RCN1_HUMAN	39	8/8	21/12	2.27	0.0026
Tropomodulin-3	TMOD3_HUMAN	40	4/4	9/6	2.24	0.0029
Discoidin, CUB and LCCL domain-containing protein 1	DCBD1_HUMAN	78	5/3	10/6	2.26	0.0032
Neuronal growth regulator 1	NEGR1_HUMAN	39	2/1	5/3	3.28	0.0033
Pregnancy-specific beta-1-glycoprotein 1	PSG1_HUMAN	47	6/1	28/8	7.31	0.0036
Transmembrane glycoprotein NMB	GPNMB_HUMAN	64	3/2	11/3	4.53	0.0037
Proteasome subunit alpha type-3	PSA3_HUMAN	28	4/3	13/7	3.35	0.0038
Nucleoside diphosphate kinase A	NDKA_HUMAN	17	12/7	32/9	2.67	0.0039
Nuclease-sensitive element-binding protein 1	YBOX1_HUMAN	36	4/2	12/5	4.58	0.0039
Transforming growth factor beta-2	TGFB2_HUMAN	48	3/3	7/4	2.40	0.0045
Dermcidin	DCD_HUMAN	11	2/1	6/3	3.50	0.0045
Scavenger receptor class F member 2	SREC2_HUMAN	92	2/2	9/6	4.30	0.0051
Phosphoglycolate phosphatase	PGP_HUMAN	34	3/3	7/7	2.96	0.0053
Dystroglycan	DAG1_HUMAN	97	12/9	28/14	2.01	0.0076
F-actin-capping protein subunit alpha-1	CAZA1_HUMAN	33	6/2	12/10	2.37	0.0078
Collagen alpha-1(II) chain	CO2A1_HUMAN	142	6/1	17/4	6.85	0.0078
Translin	TSN_HUMAN	26	7/6	15/10	2.07	0.0081
F-actin-capping protein subunit alpha-2	CAZA2_HUMAN	33	4/2	12/10	2.55	0.0082
Sialate O-acetylesterase	SIAE_HUMAN	58	4/3	7/3	2.22	0.010
Single-stranded DNA-binding protein, mitochondrial	SSBP_HUMAN	17	3/2	7/4	3.52	0.011
Dynactin subunit 1	DCTN1_HUMAN	142	2/2	5/3	3.30	0.012
Alpha/beta hydrolase domain-containing protein 14B	ABHEB_HUMAN	22	3/2	7/4	3.19	0.014
Polypeptide N-acetylgalactosaminyltransferase 5	GALT5_HUMAN	106	2/2	16/10	7.14	0.014
Aminopeptidase N	AMPN_HUMAN	110	16/13	38/20	2.32	0.016
Dynein light chain 2, cytoplasmic	DYL2_HUMAN	10	3/3	7/1	4.27	0.016
45 kDa calcium-binding protein	CAB45_HUMAN	42	4/3	9/7	2.12	0.018
Cysteine-rich motor neuron 1 protein	CRIM1_HUMAN	114	3/2	8/5	2.18	0.023
Beta-1,4-galactosyltransferase 1	B4GT1_HUMAN	44	2/2	7/5	3.50	0.024
Microtubule-associated protein 4	MAP4_HUMAN	121	2/2	6/2	2.61	0.032

Pro-neuregulin-1, membrane-bound isoform	NRG1_HUMAN	70	2/1	5/3	2.43	0.032
Ribonuclease UK114	UK114_HUMAN	14	2/1	3/3	2.84	0.034
Collagen triple helix repeat-containing protein 1	CTHR1_HUMAN	26	2/2	3/3	2.79	0.035
Ubiquitin-conjugating enzyme E2 variant 2	UB2V2_HUMAN	16	5/1	9/5	2.66	0.041
Protein S100-A16	S10AG_HUMAN	12	4/3	6/4	2.14	0.043
Peptidyl-prolyl cis-trans isomerase FKBP10	FKB10_HUMAN	64	2/2	8/7	4.23	0.048

Proteins up-regulated in SEN ESCs secretome, quantified with at least 1 spectral counts (sc)

Identified Proteins	Uniprot ID	MW (kDa)	Condition (max # sc/peptides) Ctrl	Sen	Ratio Sen/Ctrl	*p-value*
Talin-1	TLN1_HUMAN	270	1/1	54/37	147.94	< 0.00010
Integrin beta-5	ITB5_HUMAN	88	1/1	12/9	35.75	< 0.00010
Delta-aminolevulinic acid dehydratase	HEM2_HUMAN	36	1/1	7/6	5.91	< 0.00010
Myosin regulatory light chain 12A	ML12A_HUMAN	20	1/1	6/5	5.28	< 0.00010
Calpain small subunit 1	CPNS1_HUMAN	28	1/1	5/4	4.04	0.00038
Clusterin	CLUS_HUMAN	52	1/1	6/5	7.55	0.00053
Importin subunit beta-1	IMB1_HUMAN	97	1/1	9/7	22.83	0.00067
Proteasome subunit alpha type-5	PSA5_HUMAN	26	1/1	10/6	24.52	0.00077
Integrin alpha-5	ITA5_HUMAN	115	1/1	5/3	6.60	0.00082
Cell adhesion molecule 4	CADM4_HUMAN	43	1/1	5/4	12.23	0.001
Nucleotide exchange factor SIL1	SIL1_HUMAN	52	1/1	8/7	6.55	0.001
Heat shock protein beta-1	HSPB1_HUMAN	23	1/1	8/3	19.20	0.0011
Granulins	GRN_HUMAN	64	1/1	8/4	10.21	0.0013
Stanniocalcin-1	STC1_HUMAN	28	1/1	7/4	8.34	0.0015
Receptor-type tyrosine-protein phosphatase mu	PTPRM_HUMAN	164	1/1	10/8	12.92	0.0016
5'-nucleotidase	5NTD_HUMAN	63	1/1	7/4	15.98	0.0018
Midkine	MK_HUMAN	16	1/1	8/5	6.50	0.0027
Guanylate kinase	KGUA_HUMAN	22	1/1	8/4	18.85	0.0028
Lysosome-associated membrane glycoprotein 1	LAMP1_HUMAN	45	1/1	4/3	10.45	0.0029
Carboxypeptidase D	CBPD_HUMAN	153	1/1	3/3	4.25	0.003
High mobility group protein B1	HMGB1_HUMAN	25	1/1	4/3	4.72	0.005
Cadherin-6	CADH6_HUMAN	88	1/1	6/5	6.49	0.0055
Thioredoxin	THIO_HUMAN	12	1/1	3/3	2.48	0.0056

Perilipin-3	PLIN3_HUMAN	47	1/1	8/3	8.48	0.0062
Endoplasmic reticulum aminopeptidase 2	ERAP2_HUMAN	110	1/1	3/3	7.50	0.0074
Contactin-3	CNTN3_HUMAN	113	1/1	4/4	4.67	0.008
Cystatin-M	CYTM_HUMAN	17	1/1	3/2	7.60	0.0098
Proteasome activator complex subunit 1	PSME1_HUMAN	29	1/1	3/3	7.31	0.011
Density-regulated protein	DENR_HUMAN	22	1/1	3/3	7.31	0.011
Ovarian cancer-associated gene 2 protein	OVCA2_HUMAN	24	1/1	2/2	5.47	0.011
Macrophage colony-stimulating factor 1	CSF1_HUMAN	60	1/1	6/5	4.33	0.012
Spectrin alpha chain, non-erythrocytic 1	SPTN1_HUMAN	285	1/1	3/2	3.76	0.013
CD59 glycoprotein	CD59_HUMAN	14	1/1	3/2	6.37	0.015
Thrombospondin type-1 domain-containing protein 4	THSD4_HUMAN	112	1/1	4/3	8.53	0.017
A disintegrin and metalloproteinase with thrombospondin motifs 7	ATS7_HUMAN	184	1/1	3/3	3.79	0.017
Acyl-CoA-binding protein	ACBP_HUMAN	10	1/1	2/2	2.84	0.022
Pleiotrophin	PTN_HUMAN	19	1/1	4/3	2.78	0.024
Zyxin	ZYX_HUMAN	61	1/1	5/3	9.35	0.032
