# Supplementary material for: Molecular basis of senescence transmitting in the population of human endometrial stromal cells
Source: Aging (Albany NY). 2019 Nov 5;11(21):9912–31. doi: 10.18632/aging.102441 (PMC6874437; doi:10.18632/aging.102441)
Supplement: Supplementary Table 4 [file aging-11-102441-s005.pdf]

Supplementary Table 4. Proteins down-regulated in SEN ESCs secretome, quantified with at least 2 spectral counts (sc)

Identified Proteins	Uniprot ID	MW (kDa)	Condition (max # sc/peptides)		Ratio Sen/Ctrl	*p-value*
			Ctrl	Sen		
Xaa-Pro dipeptidase	PEPD_HUMAN	55	39/14	3/2	0.05	< 0.00010
Soluble calcium-activated nucleotidase 1	CANT1_HUMAN	45	19/12	2/2	0.05	< 0.00010
Filamin-B	FLNB_HUMAN	278	138/61	10/1	0.07	< 0.00010
Lactotransferrin	TRFL_HUMAN	78	109/34	10/3	0.09	< 0.00010
Fascin	FSCN1_HUMAN	55	59/20	8/5	0.10	< 0.00010
Dihydrolipoyl dehydrogenase, mitochondrial	DLDH_HUMAN	54	26/12	3/3	0.11	< 0.00010
Procollagen-lysine,2-oxoglutarate 5-dioxygenase 3	PLOD3_HUMAN	85	22/15	3/3	0.11	< 0.00010
Serum albumin	ALBU_HUMAN	69	278/45	34/5	0.12	< 0.00010
Interstitial collagenase	MMP1_HUMAN	54	80/18	13/6	0.12	< 0.00010
WD repeat-containing protein 1	WDR1_HUMAN	66	42/20	5/3	0.12	< 0.00010
Serpin B7	SPB7_HUMAN	43	24/12	5/3	0.12	< 0.00010
Lysozyme C	LYSC_HUMAN	17	12/5	2/1	0.12	< 0.00010
Transketolase	TKT_HUMAN	68	104/24	17/6	0.15	< 0.00010
Glutathione synthetase	GSHB_HUMAN	52	28/12	5/3	0.15	< 0.00010
Dihydropyrimidinase-related protein 2	DPYL2_HUMAN	62	14/11	2/2	0.15	< 0.00010
Secernin-1	SCRN1_HUMAN	46	11/6	2/2	0.15	< 0.00010
Heat shock 70 kDa protein 4	HSP74_HUMAN	94	33/21	7/5	0.18	< 0.00010
Filamin-A	FLNA_HUMAN	281	227/71	63/33	0.27	< 0.00010
Filamin-C	FLNC_HUMAN	291	144/49	39/18	0.27	< 0.00010
Nidogen-1	NID1_HUMAN	136	88/28	26/14	0.28	< 0.00010
Puromycin-sensitive aminopeptidase	PSA_HUMAN	103	56/29	15/9	0.28	< 0.00010
Cathepsin L1	CATL1_HUMAN	38	20/7	6/1	0.29	< 0.00010
Thioredoxin reductase 1, cytoplasmic	TRXR1_HUMAN	71	116/27	35/13	0.31	< 0.00010
Rab GDP dissociation inhibitor alpha	GDIA_HUMAN	51	67/15	24/4	0.36	< 0.00010
Purine nucleoside phosphorylase	PNPH_HUMAN	32	39/16	16/10	0.38	< 0.00010
Calsyntenin-1	CSTN1_HUMAN	110	113/32	46/16	0.40	< 0.00010
Tyrosine-protein kinase receptor UFO	UFO_HUMAN	98	24/5	10/5	0.40	< 0.00010

Collagen alpha-3(VI) chain	CO6A3_HUMAN	344	314/64	129/62	0.41	< 0.00010
Collagen alpha-1(XII) chain	COCA1_HUMAN	333	210/99	90/57	0.41	< 0.00010
78 kDa glucose-regulated protein	GRP78_HUMAN	72	82/25	39/18	0.47	< 0.00010
Peroxidasin homolog	PXDN_HUMAN	165	33/19	16/7	0.47	< 0.00010
Keratin, type II cytoskeletal 5	K2C5_HUMAN	62	41/7	18/4	0.43	0.0001
Keratin, type I cytoskeletal 14	K1C14_HUMAN	52	40/12	19/5	0.47	0.00014
Aldose reductase	ALDR_HUMAN	36	15/6	2/1	0.14	0.00016
Keratin, type II cytoskeletal 6C	K2C6C_HUMAN	60	35/11	17/2	0.49	0.00021
Laminin subunit alpha-5	LAMA5_HUMAN	400	14/11	4/3	0.20	0.00021
Dihydropteridine reductase	DHPR_HUMAN	26	14/7	6/3	0.36	0.00023
Serpin B6	SPB6_HUMAN	43	50/16	17/7	0.32	0.00025
Actin-related protein 2/3 complex subunit 1B	ARC1B_HUMAN	41	12/4	4/1	0.28	0.00033
Cytosol aminopeptidase	AMPL_HUMAN	56	26/16	9/7	0.30	0.00035
Thyrotropin-releasing hormone-degrading ectoenzyme	TRHDE_HUMAN	117	24/15	6/4	0.23	0.00036
Serpin H1	SERPH_HUMAN	46	16/11	6/3	0.35	0.00036
Serine/threonine-protein phosphatase PP1-alpha catalytic subunit	PP1A_HUMAN	38	38/14	6/6	0.06	0.00039
Actin-related protein 2/3 complex subunit 2	ARPC2_HUMAN	34	28/14	2/2	0.07	0.0004
Transcobalamin-2	TCO2_HUMAN	48	15/9	4/3	0.13	0.00044
Heat shock 70 kDa protein 1A/1B	HSP71_HUMAN	70	26/13	10/3	0.35	0.00045
Prolyl endopeptidase FAP	SEPR_HUMAN	88	12/7	5/3	0.35	0.00049
Neuropilin-2	NRP2_HUMAN	105	25/16	11/6	0.46	0.00067
Putative GTP cyclohydrolase 1 type 2 NIF3L1	GTPC1_HUMAN	42	9/6	2/2	0.20	0.00068
Actin-related protein 3	ARP3_HUMAN	47	31/17	15/8	0.45	0.00071
Keratin, type I cytoskeletal 16	K1C16_HUMAN	51	37/4	17/1	0.44	0.00078
Moesin	MOES_HUMAN	68	62/21	9/5	0.10	0.00079
Prolactin-inducible protein	PIP_HUMAN	17	11/6	3/3	0.15	0.00089
Growth arrest-specific protein 6	GAS6_HUMAN	80	13/7	7/5	0.47	0.0011
Acylpyruvase FAHD1, mitochondrial	FAHD1_HUMAN	25	12/4	4/3	0.27	0.0012
Actin-related protein 2	ARP2_HUMAN	45	18/9	7/4	0.22	0.0014
S-formylglutathione hydrolase	ESTD_HUMAN	31	6/6	2/2	0.17	0.0014
AP-2 complex subunit beta	AP2B1_HUMAN	105	5/4	2/2	0.43	0.0015
Rab GDP dissociation inhibitor beta	GDIB_HUMAN	51	71/25	35/10	0.46	0.0016

Serine/threonine-protein phosphatase PP1-beta catalytic subunit	PP1B_HUMAN	37	35/4	8/7	0.14	0.0016
Glyoxalase domain-containing protein 4	GLOD4_HUMAN	35	16/9	10/7	0.47	0.0019
Serine/threonine-protein phosphatase CPPED1	CPPED_HUMAN	36	16/10	6/5	0.30	0.0022
Dipeptidyl peptidase 1	CATC_HUMAN	52	22/8	5/3	0.20	0.0022
Dipeptidyl peptidase 3	DPP3_HUMAN	83	42/22	13/10	0.32	0.0027
Hemoglobin subunit beta	HBB_HUMAN	16	6/3	2/2	0.22	0.0027
Hydroxyacylglutathione hydrolase, mitochondrial	GLO2_HUMAN	34	8/6	5/3	0.48	0.0031
EGF-containing fibulin-like extracellular matrix protein 2	FBLN4_HUMAN	49	14/10	5/4	0.25	0.0032
Coronin-1C	COR1C_HUMAN	53	10/7	2/2	0.15	0.0036
Peptidyl-prolyl cis-trans isomerase-like 3	PPIL3_HUMAN	18	9/6	5/4	0.46	0.0046
Ribosyldihydronicotinamide dehydrogenase [quinone]	NQO2_HUMAN	26	10/6	4/4	0.27	0.005
Chitinase domain-containing protein 1	CHID1_HUMAN	45	26/13	11/7	0.44	0.0051
3'(2'),5'-bisphosphate nucleotidase 1	BPNT1_HUMAN	33	10/9	4/4	0.43	0.0051
Fumarylacetoacetase	FAAA_HUMAN	46	12/9	6/6	0.34	0.0058
Proteasome subunit alpha type-6	PSA6_HUMAN	27	6/5	3/2	0.29	0.0064
Transitional endoplasmic reticulum ATPase	TERA_HUMAN	89	15/11	7/5	0.42	0.0089
Adenylosuccinate synthetase isozyme 2	PURA2_HUMAN	50	6/5	3/3	0.29	0.013
Tumor necrosis factor receptor superfamily member 27	TNR27_HUMAN	33	4/3	2/2	0.46	0.013
60S ribosomal protein L10a	RL10A_HUMAN	25	20/6	5/5	0.24	0.014
NADP-dependent malic enzyme	MAOX_HUMAN	64	15/11	8/5	0.43	0.015
Ras-related protein Rab-7a	RAB7A_HUMAN	23	15/9	2/2	0.16	0.017
Proteasome subunit alpha type-7	PSA7_HUMAN	28	5/5	2/2	0.21	0.017
40S ribosomal protein SA	RSSA_HUMAN	33	3/3	2/1	0.31	0.017
Proteasome subunit beta type-1	PSB1_HUMAN	26	11/8	7/6	0.40	0.02
Cathepsin K	CATK_HUMAN	37	6/4	2/2	0.20	0.022
Protocadherin-10	PCD10_HUMAN	113	9/8	4/4	0.49	0.026
Adenylyl cyclase-associated protein 1	CAP1_HUMAN	52	13/8	7/4	0.50	0.033
Angiogenin	ANGI_HUMAN	17	9/3	5/3	0.47	0.033

Disintegrin and metalloproteinase domain-containing protein 19	ADA19_HUMAN	105	7/4	5/3	0.46	0.036

Proteins down-regulated in SEN ESCs secretome, quantified with at least 1 spectral counts (sc)

**Identified Proteins**	**Uniprot ID**	**MW (kDa)**	**Condition (max # sc/peptides)**		**Ratio Sen/Ctrl**	***p-value***
			**Ctrl**	**Sen**		
Leukocyte elastase inhibitor	ILEU_HUMAN	43	24/13	1/1	0.01	0.00019
Bleomycin hydrolase	BLMH_HUMAN	53	7/3	1/1	0.09	0.00023
Inositol monophosphatase 1	IMPA1_HUMAN	30	12/10	1/1	0.06	0.00024
Intraflagellar transport protein 25 homolog	IFT25_HUMAN	16	6/5	1/1	0.05	0.00035
Enolase-phosphatase E1	ENOPH_HUMAN	29	11/7	1/1	0.04	0.00058
Omega-amidase NIT2	NIT2_HUMAN	31	7/7	1/1	0.05	0.00068
Gamma-glutamylcyclotransferase	GGCT_HUMAN	21	11/7	1/1	0.03	0.00088
Enoyl-CoA delta isomerase 1, mitochondrial	ECI1_HUMAN	33	3/2	1/1	0.10	0.00095
Proteasome subunit beta type-2	PSB2_HUMAN	23	5/4	1/1	0.13	0.001
Phosphoglucomutase-2	PGM2_HUMAN	68	17/10	1/1	0.02	0.0018
S-adenosylmethionine synthase isoform type-2	METK2_HUMAN	44	8/8	1/1	0.05	0.0019
Ephrin type-A receptor 5	EPHA5_HUMAN	115	4/3	1/1	0.08	0.0019
Pyridoxal kinase	PDXK_HUMAN	35	11/7	1/1	0.04	0.0039
Secreted and transmembrane protein 1	SCTM1_HUMAN	27	5/3	1/1	0.08	0.0041
Collagen alpha-1(VII) chain	CO7A1_HUMAN	295	25/12	1/1	0.02	0.0047
Destrin	DEST_HUMAN	19	8/6	1/1	0.15	0.0049
Pantetheinase	VNN1_HUMAN	57	3/2	1/1	0.35	0.0049
Hemopexin	HEMO_HUMAN	52	3/3	1/1	0.35	0.0049
Methionine adenosyltransferase 2 subunit beta	MAT2B_HUMAN	38	6/5	1/1	0.20	0.0068
Platelet-activating factor acetylhydrolase IB subunit alpha	LIS1_HUMAN	47	3/2	1/1	0.12	0.0076
EMILIN-2	EMIL2_HUMAN	116	3/2	1/1	0.12	0.0079
Spectrin beta chain, non-erythrocytic 1	SPTB2_HUMAN	275	3/3	1/1	0.40	0.011
Selenoprotein M	SELM_HUMAN	16	2/2	1/1	0.31	0.012

D-tyrosyl-tRNA(Tyr) deacylase 1	DTD1_HUMAN	23	3/2	1/1	0.26	0.017
Elongation factor 2	EF2_HUMAN	95	4/3	1/1	0.11	0.018
Ubiquitin-like modifier-activating enzyme 1	UBA1_HUMAN	118	2/2	1/1	0.19	0.042
